# Supplementary material for: Endosomal trafficking of two-pore K+ efflux channel TWIK2 to plasmalemma mediates NLRP3 inflammasome activation and inflammatory injury
Source: eLife. 2023 May 9;12:e83842. doi: 10.7554/eLife.83842 (PMC10202452; doi:10.7554/eLife.83842)
Supplement: Figure 3—source data 1. — Related to Figure 3C. Inhibition of vesicle–plasmalemma fusion prevents NLRP3 inflammasome activation in macrophages. (C) Representative results of western blot from three independent experiments showing reduced caspase 1 activation (reduced Casp-1 p20, C). Monocyte-derived macrophages (MDMs) pretreated with vesicle–plasmalemma fusion inhibitor Vacuolin (10 µM, 2 hr) were primed with lipopolysaccharide (LPS; 3 hr) and subsequently challenged with ATP (5 mM) for 30 min. Cell lysates or pellets were immunoblotted with indicated antibodies (anti-TWIK2 or anti-IL1β). [file elife-83842-fig3-data1.zip › Figure 3 - source data 1/Figure 3.pptx]

## Slide 1
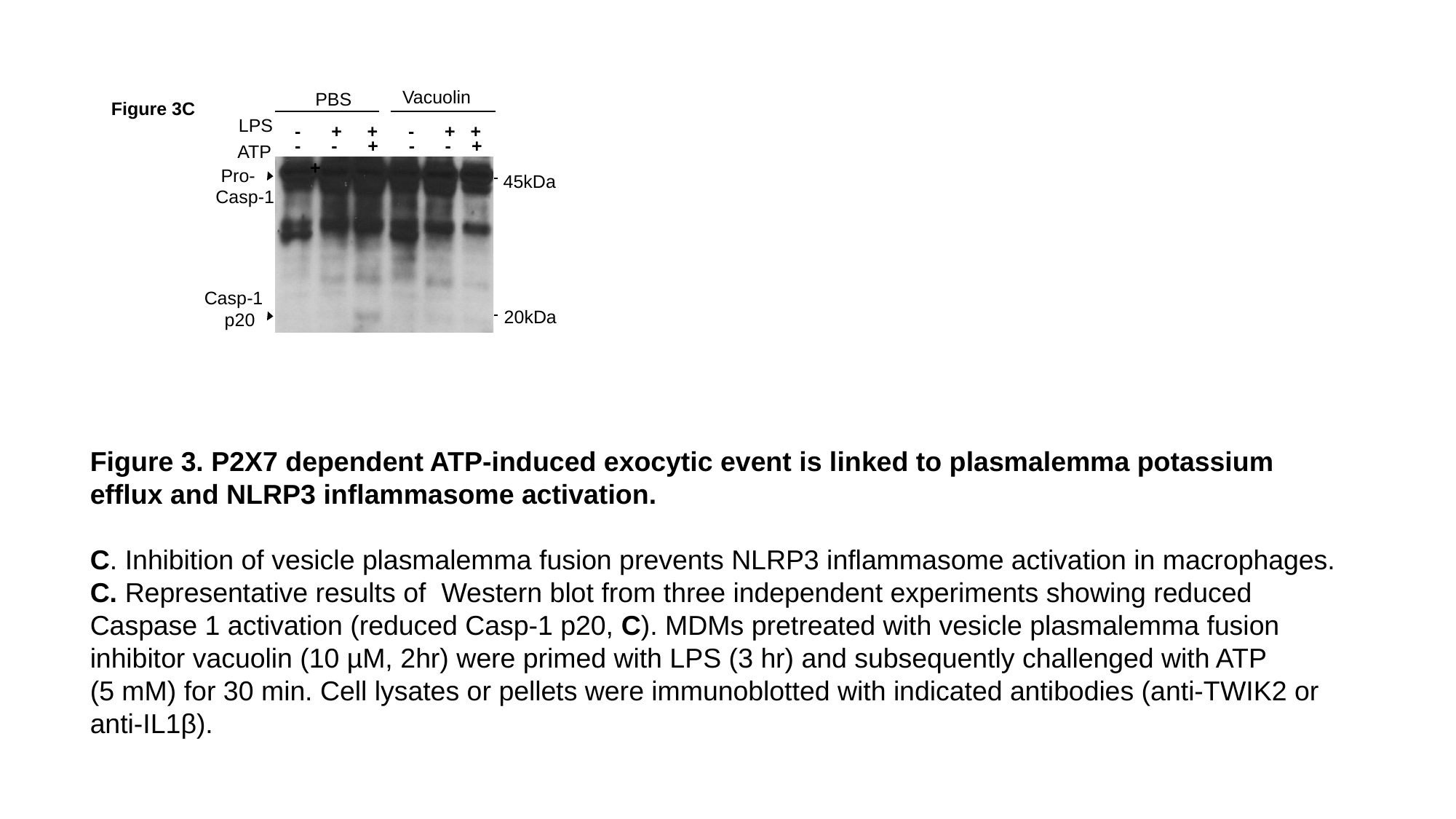

Vacuolin
PBS
LPS
- + + - + +
- - + - - + +
ATP
 Pro-
Casp-1
45kDa
 Casp-1
 p20
20kDa
Figure 3C
Figure 3. P2X7 dependent ATP-induced exocytic event is linked to plasmalemma potassium efflux and NLRP3 inflammasome activation.
C. Inhibition of vesicle plasmalemma fusion prevents NLRP3 inflammasome activation in macrophages. C. Representative results of Western blot from three independent experiments showing reduced Caspase 1 activation (reduced Casp-1 p20, C). MDMs pretreated with vesicle plasmalemma fusion inhibitor vacuolin (10 µM, 2hr) were primed with LPS (3 hr) and subsequently challenged with ATP (5 mM) for 30 min. Cell lysates or pellets were immunoblotted with indicated antibodies (anti-TWIK2 or anti-IL1β).
