# Supplementary figures and images for: Endosomal trafficking of two-pore K+ efflux channel TWIK2 to plasmalemma mediates NLRP3 inflammasome activation and inflammatory injury
Source: eLife. 2023 May 9;12:e83842. doi: 10.7554/eLife.83842 (PMC10202452; doi:10.7554/eLife.83842)

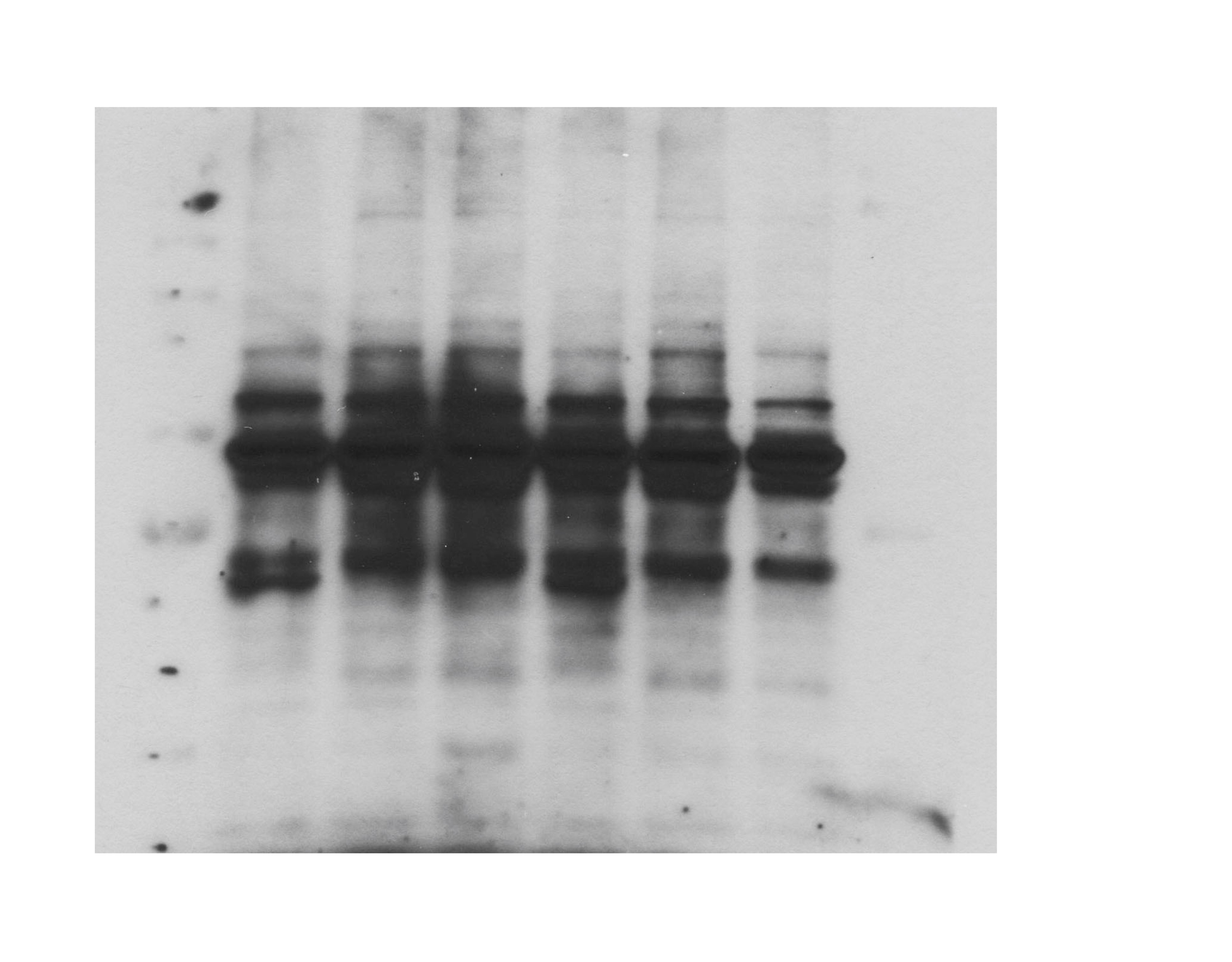

Supplement: Figure 3—source data 1. — Related to Figure 3C. Inhibition of vesicle–plasmalemma fusion prevents NLRP3 inflammasome activation in macrophages. (C) Representative results of western blot from three independent experiments showing reduced caspase 1 activation (reduced Casp-1 p20, C). Monocyte-derived macrophages (MDMs) pretreated with vesicle–plasmalemma fusion inhibitor Vacuolin (10 µM, 2 hr) were primed with lipopolysaccharide (LPS; 3 hr) and subsequently challenged with ATP (5 mM) for 30 min. Cell lysates or pellets were immunoblotted with indicated antibodies (anti-TWIK2 or anti-IL1β). [file elife-83842-fig3-data1.zip › Figure 3 - source data 1/Figure 3 - source data 1 for original WB.jpg]

## Slide 1
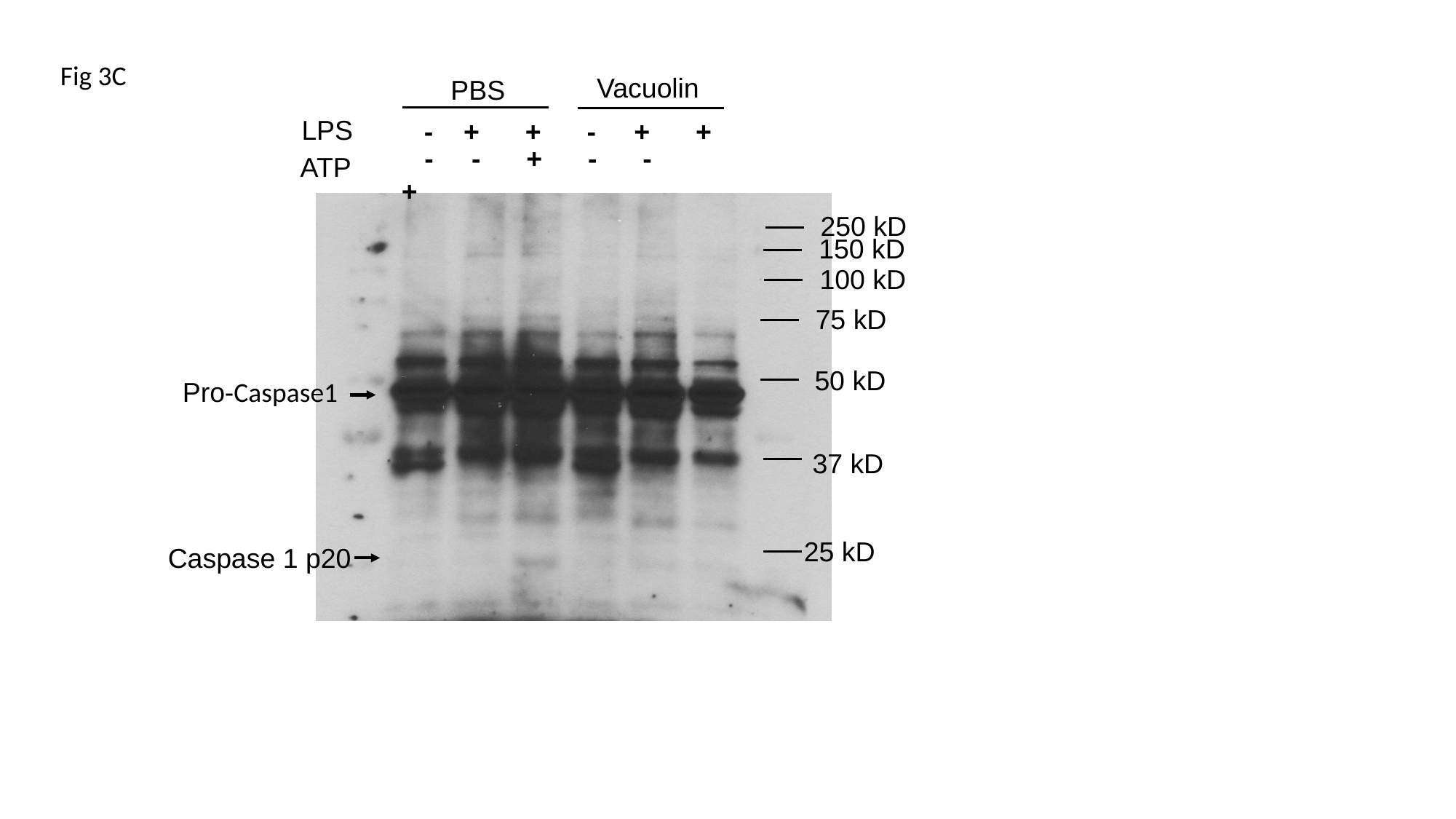

Fig 3C
Vacuolin
PBS
LPS
 - + + - + +
 - - + - - +
ATP
250 kD
150 kD
100 kD
75 kD
50 kD
Pro-Caspase1
37 kD
25 kD
 Caspase 1 p20

Supplement: Figure 3—source data 1. — Related to Figure 3C. Inhibition of vesicle–plasmalemma fusion prevents NLRP3 inflammasome activation in macrophages. (C) Representative results of western blot from three independent experiments showing reduced caspase 1 activation (reduced Casp-1 p20, C). Monocyte-derived macrophages (MDMs) pretreated with vesicle–plasmalemma fusion inhibitor Vacuolin (10 µM, 2 hr) were primed with lipopolysaccharide (LPS; 3 hr) and subsequently challenged with ATP (5 mM) for 30 min. Cell lysates or pellets were immunoblotted with indicated antibodies (anti-TWIK2 or anti-IL1β). [file elife-83842-fig3-data1.zip › Figure 3 - source data 1/Figure 3 - source data 1 for WB labelled.pptx]

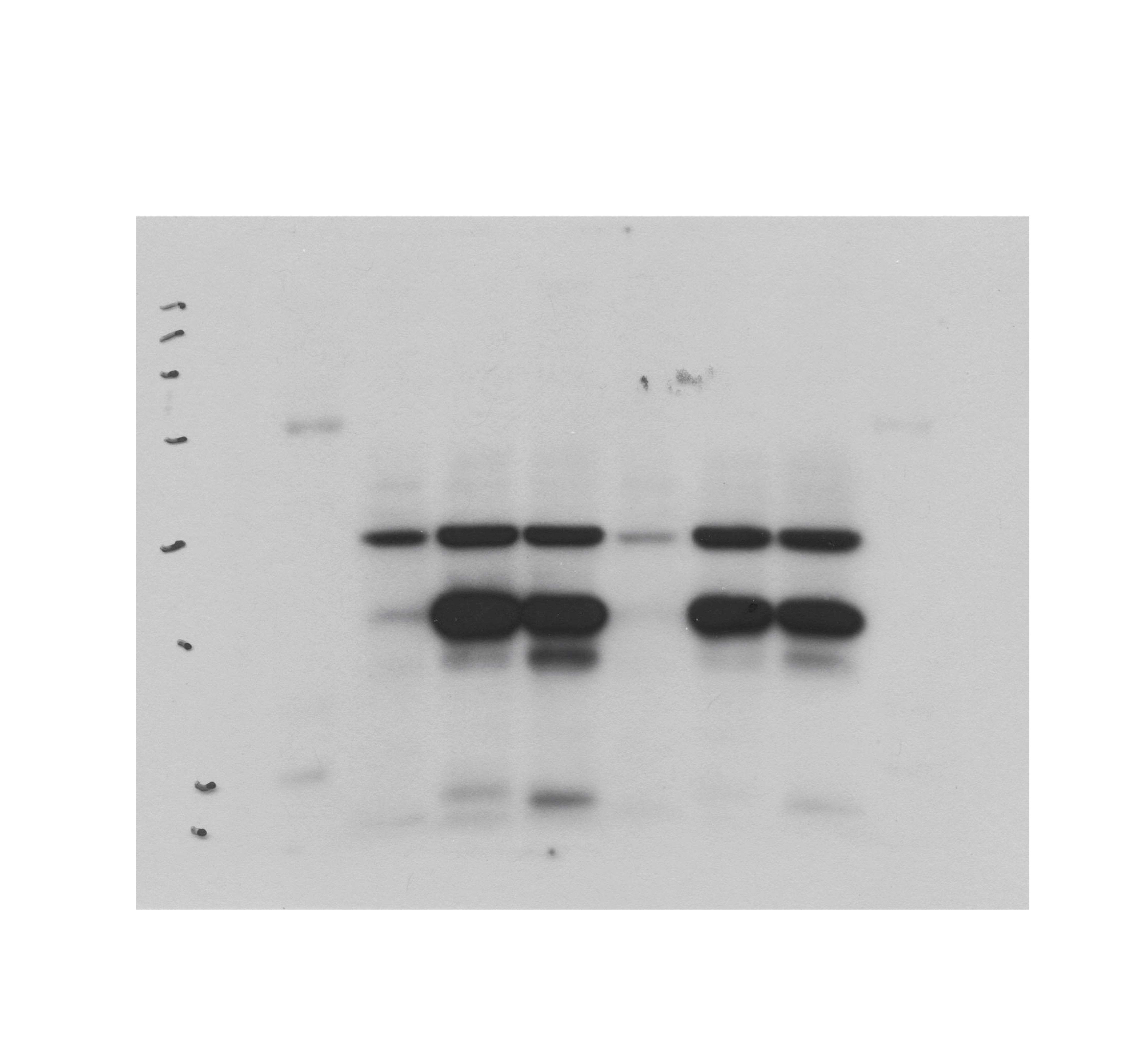

Supplement: Figure 3—source data 2. — Related to Figure 3E. Representative results of western blot from three independent experiments showing reduced IL-1β maturation (reduced IL-1β p17). Monocyte-derived macrophages (MDMs) pretreated with vesicle–plasmalemma fusion inhibitor Vacuolin (10 µM, 2 hr) were primed with lipopolysaccharide (LPS; 3 hr) and subsequently challenged with ATP (5 mM) for 30 min. Cell lysates or pellets were immunoblotted with indicated antibodies (anti-TWIK2 or anti-IL1β). [file elife-83842-fig3-data2.zip › Figure 3 - source data 2/Figure 3 - source data 2 for original WB IL-1b.jpg]

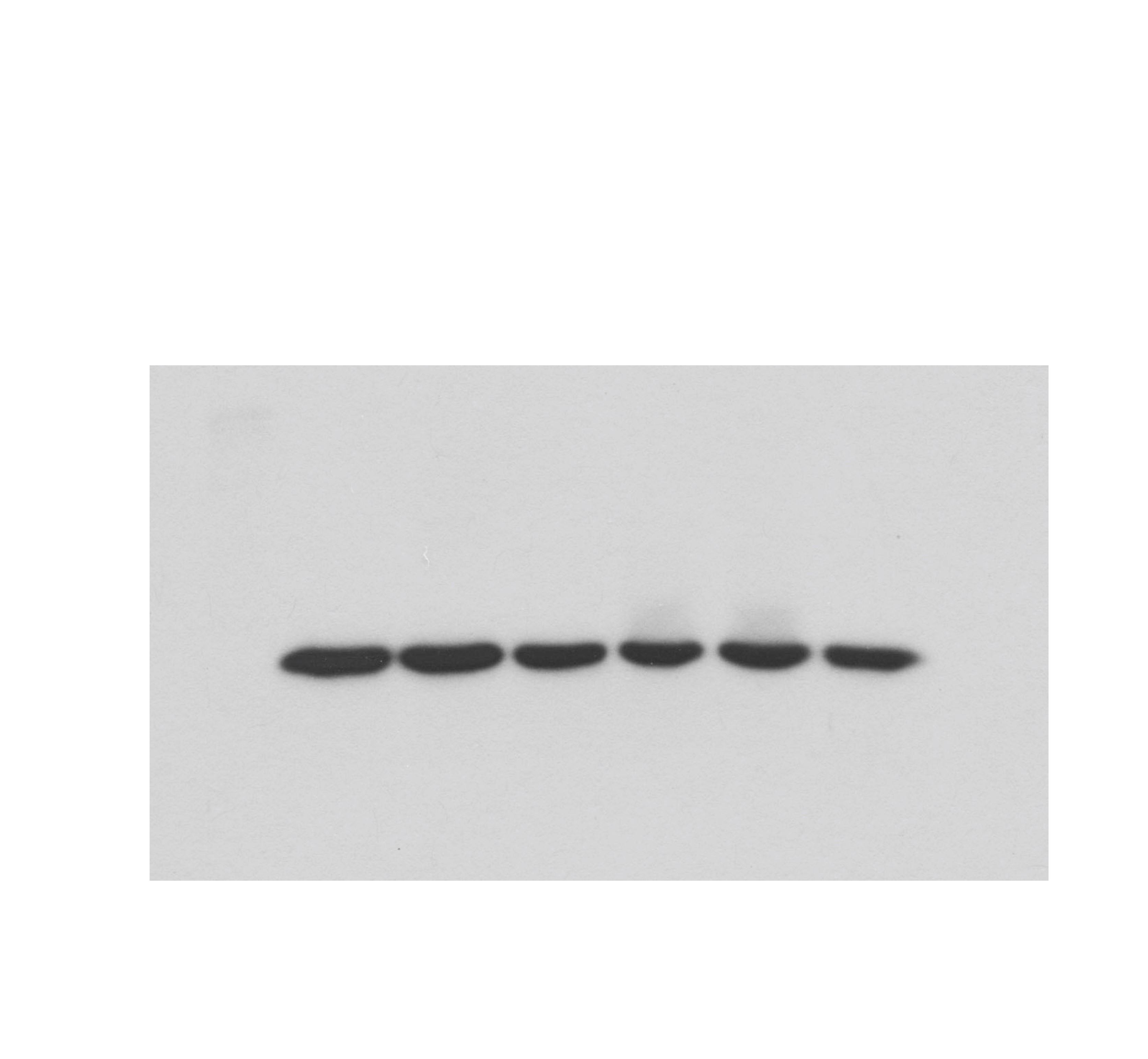

Supplement: Figure 3—source data 2. — Related to Figure 3E. Representative results of western blot from three independent experiments showing reduced IL-1β maturation (reduced IL-1β p17). Monocyte-derived macrophages (MDMs) pretreated with vesicle–plasmalemma fusion inhibitor Vacuolin (10 µM, 2 hr) were primed with lipopolysaccharide (LPS; 3 hr) and subsequently challenged with ATP (5 mM) for 30 min. Cell lysates or pellets were immunoblotted with indicated antibodies (anti-TWIK2 or anti-IL1β). [file elife-83842-fig3-data2.zip › Figure 3 - source data 2/Figure 3 - source data2 for original WB GADPH.jpg]

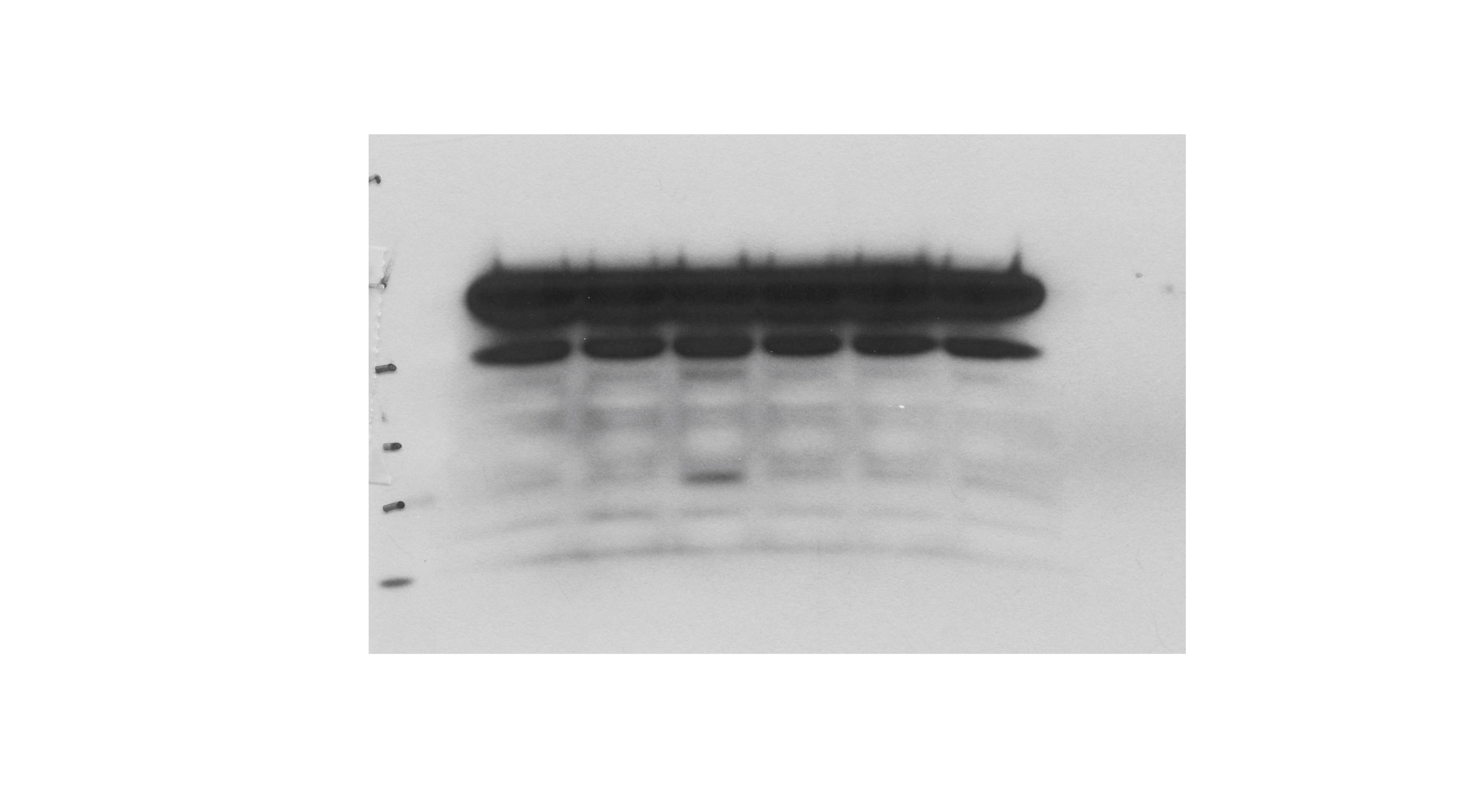

Supplement: Figure 4—source data 1. — Related to Figure 4C. Extracellular Ca2+-dependent NLRP3 inflammasome activation in macrophages. Monocyte-derived macrophages (MDMs) were primed with lipopolysaccharide (LPS) and subsequently challenged with ATP and cell lysates or pellets were immunoblotted with indicated antibodies (anti-Caspase 1 or anti-IL1β). Representative western blotting results from three independent experiments showing reduced caspase 1 activation (reduced Casp-1 p20) in the absence of extracellular Ca2+. [file elife-83842-fig4-data1.zip › Figure 4 - source data 1/Figure 4 - source data 1 for original WB.jpg]

## Slide 1
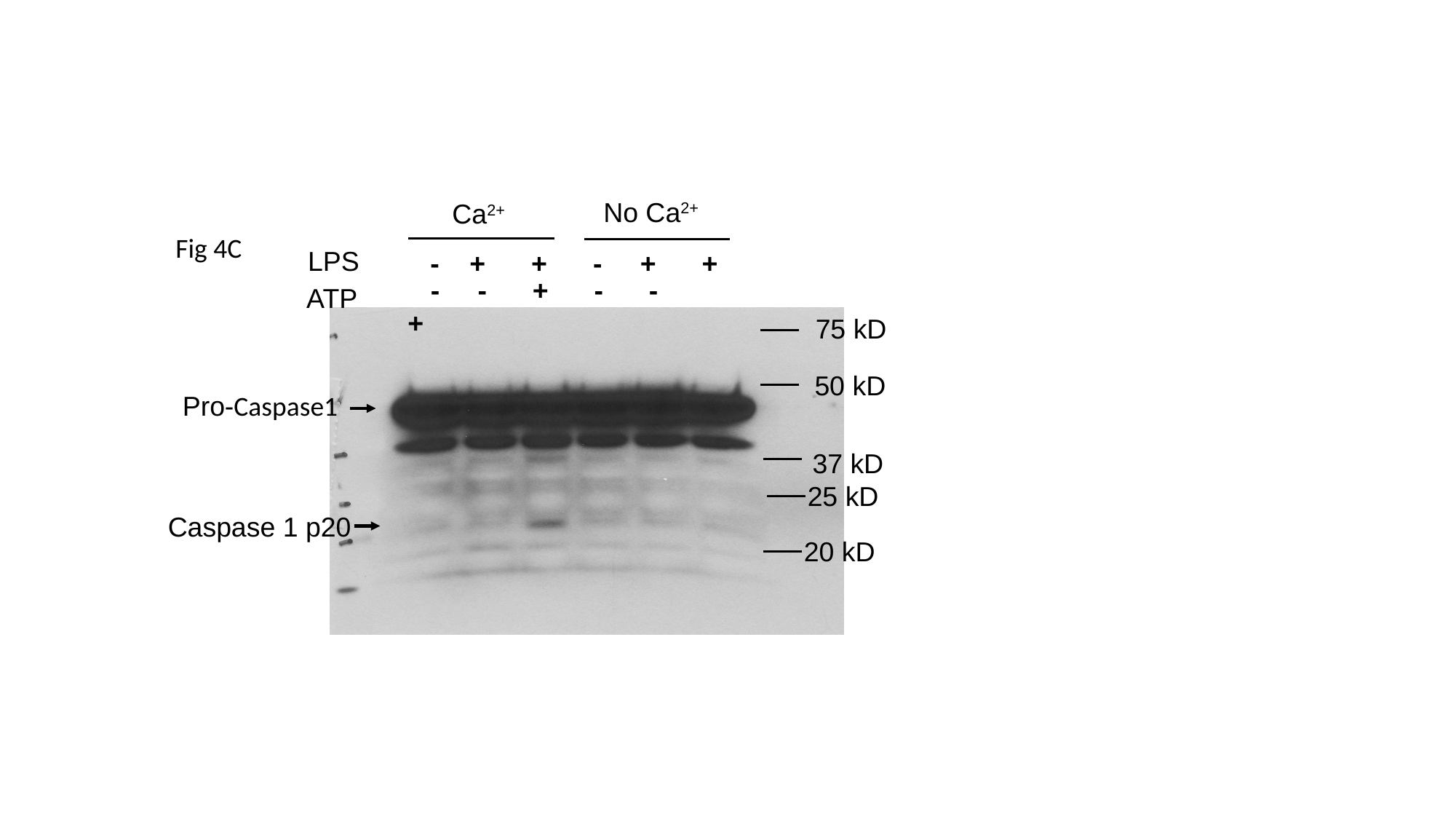

No Ca2+
Ca2+
Fig 4C
LPS
 - + + - + +
 - - + - - +
ATP
75 kD
50 kD
Pro-Caspase1
37 kD
25 kD
 Caspase 1 p20
20 kD

Supplement: Figure 4—source data 1. — Related to Figure 4C. Extracellular Ca2+-dependent NLRP3 inflammasome activation in macrophages. Monocyte-derived macrophages (MDMs) were primed with lipopolysaccharide (LPS) and subsequently challenged with ATP and cell lysates or pellets were immunoblotted with indicated antibodies (anti-Caspase 1 or anti-IL1β). Representative western blotting results from three independent experiments showing reduced caspase 1 activation (reduced Casp-1 p20) in the absence of extracellular Ca2+. [file elife-83842-fig4-data1.zip › Figure 4 - source data 1/Figure 4 - source data 1 for WB labelled.pptx]

## Slide 1
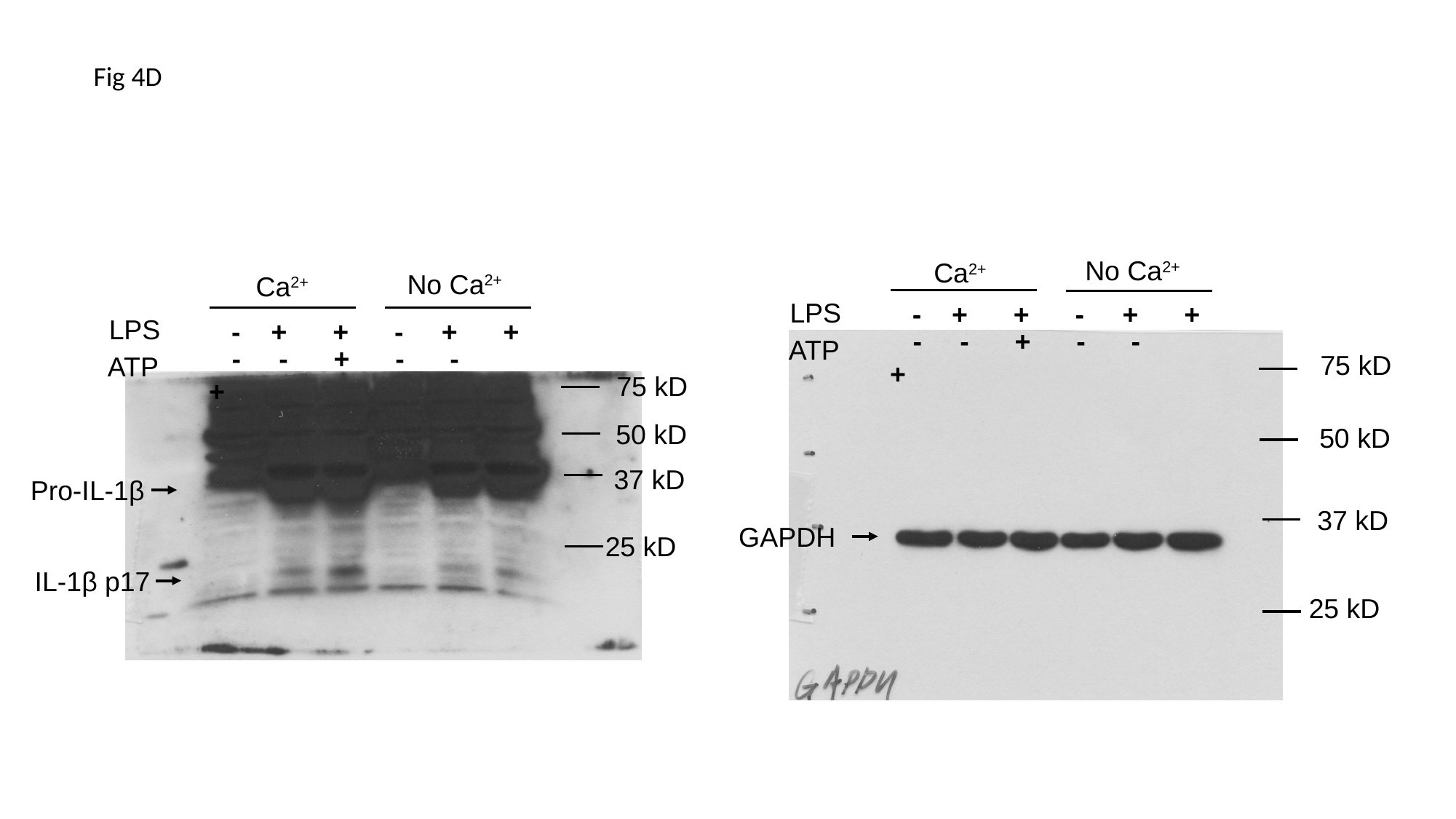

Fig 4D
No Ca2+
Ca2+
LPS
 - + + - + +
 - - + - - +
ATP
75 kD
50 kD
37 kD
GAPDH
25 kD
No Ca2+
Ca2+
LPS
 - + + - + +
 - - + - - +
ATP
75 kD
50 kD
37 kD
Pro-IL-1β
25 kD
IL-1β p17

Supplement: Figure 4—source data 2. — Related to Figure 4D. Extracellular Ca2+-dependent NLRP3 inflammasome activation in macrophages. Monocyte-derived macrophages (MDMs) were primed with lipopolysaccharide (LPS) and subsequently challenged with ATP and cell lysates or pellets were immunoblotted with indicated antibodies (anti-IL1β). Representative western blotting results from three independent experiments showing reduced caspase 1 activation (reduced Casp-1 p20) and IL-1β maturation (reduced IL-1β p17) in the absence of extracellular Ca2+. [file elife-83842-fig4-data2.zip › Figure 4 - source data 2/Figure 4 - source data 2 for WB labelled.pptx]

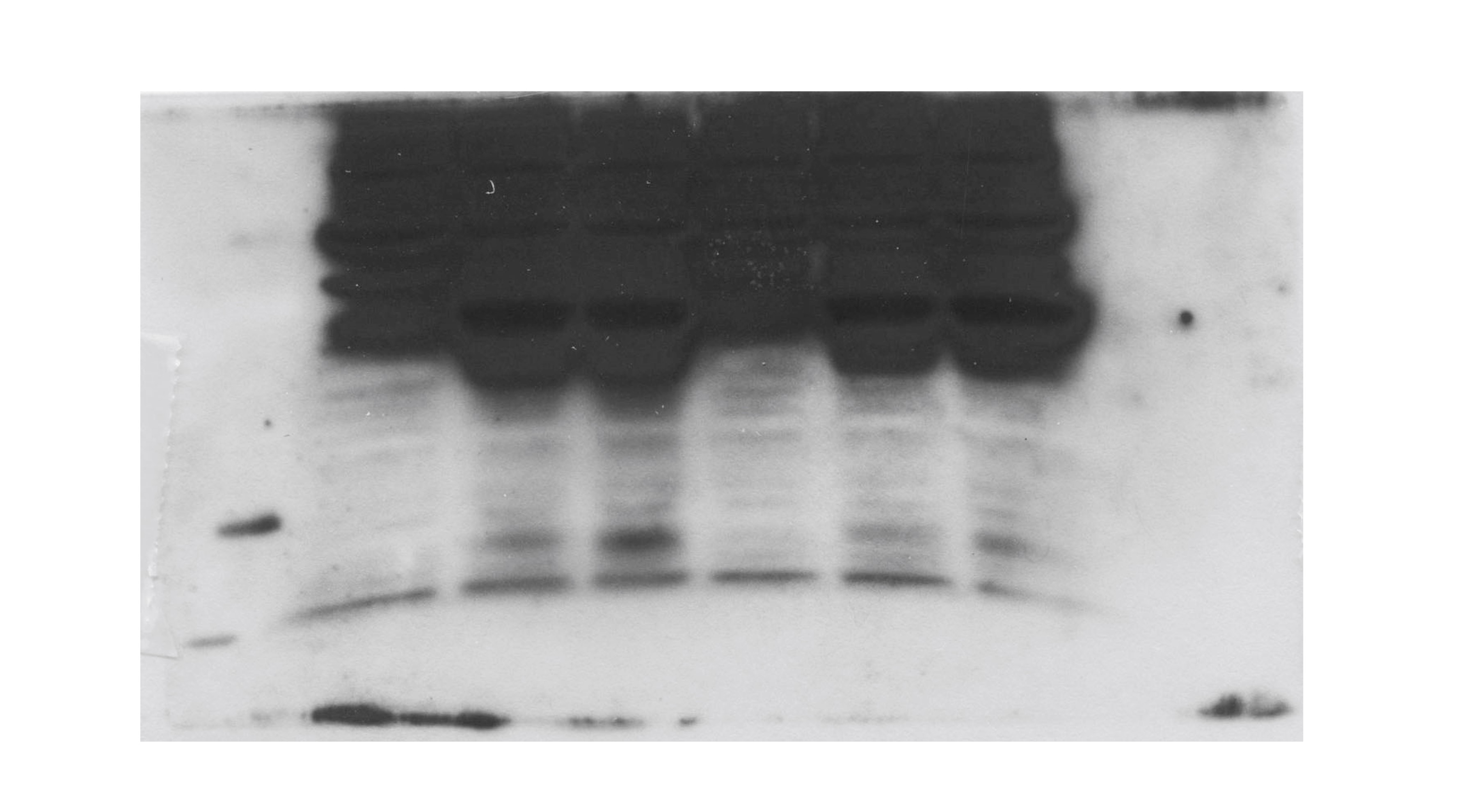

Supplement: Figure 4—source data 2. — Related to Figure 4D. Extracellular Ca2+-dependent NLRP3 inflammasome activation in macrophages. Monocyte-derived macrophages (MDMs) were primed with lipopolysaccharide (LPS) and subsequently challenged with ATP and cell lysates or pellets were immunoblotted with indicated antibodies (anti-IL1β). Representative western blotting results from three independent experiments showing reduced caspase 1 activation (reduced Casp-1 p20) and IL-1β maturation (reduced IL-1β p17) in the absence of extracellular Ca2+. [file elife-83842-fig4-data2.zip › Figure 4 - source data 2/Figure 4 - source data 2-1 for original WB IL-b.jpg]

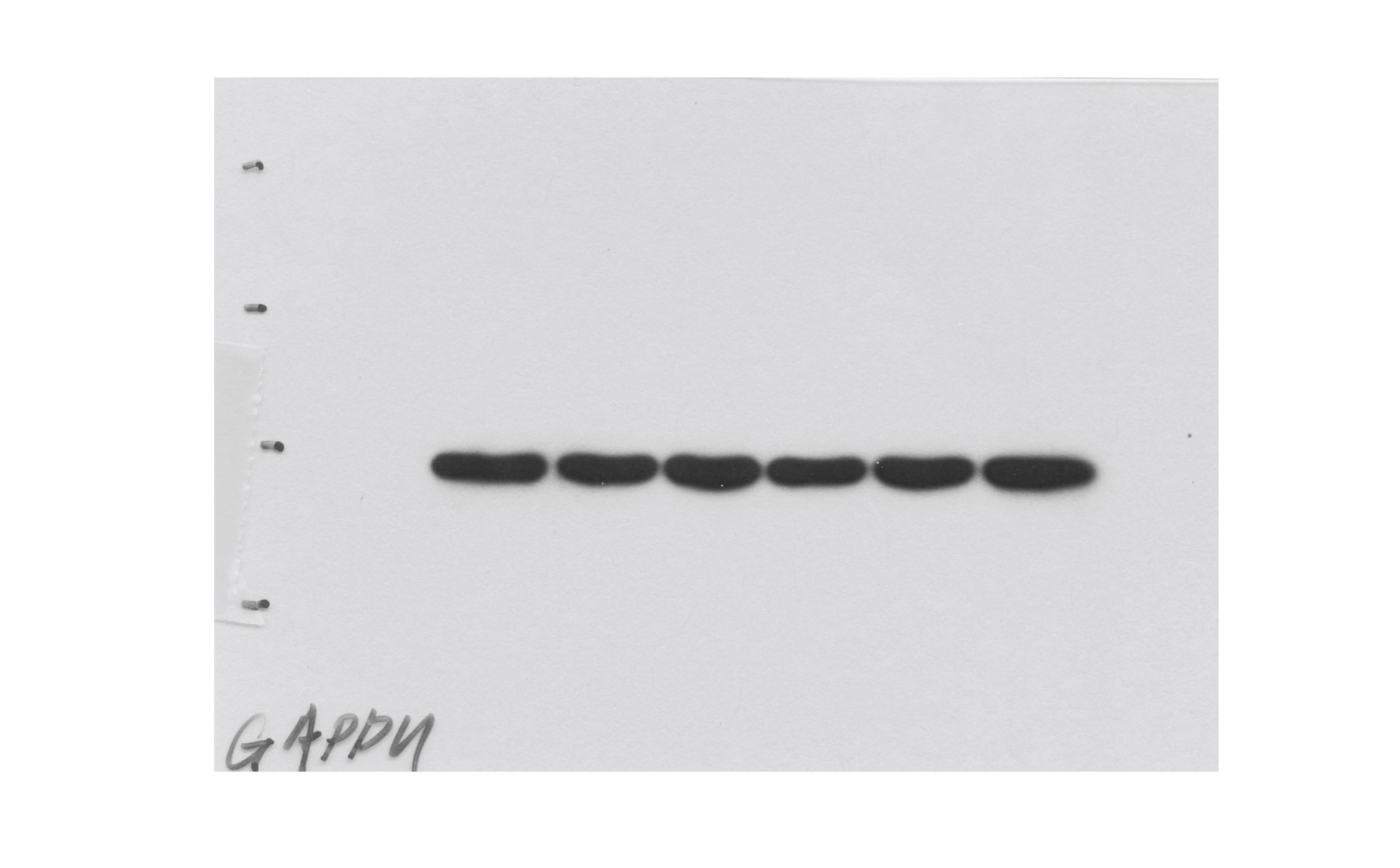

Supplement: Figure 4—source data 2. — Related to Figure 4D. Extracellular Ca2+-dependent NLRP3 inflammasome activation in macrophages. Monocyte-derived macrophages (MDMs) were primed with lipopolysaccharide (LPS) and subsequently challenged with ATP and cell lysates or pellets were immunoblotted with indicated antibodies (anti-IL1β). Representative western blotting results from three independent experiments showing reduced caspase 1 activation (reduced Casp-1 p20) and IL-1β maturation (reduced IL-1β p17) in the absence of extracellular Ca2+. [file elife-83842-fig4-data2.zip › Figure 4 - source data 2/Figure 4 - source data 2-2for original WB GAPDH.jpg]

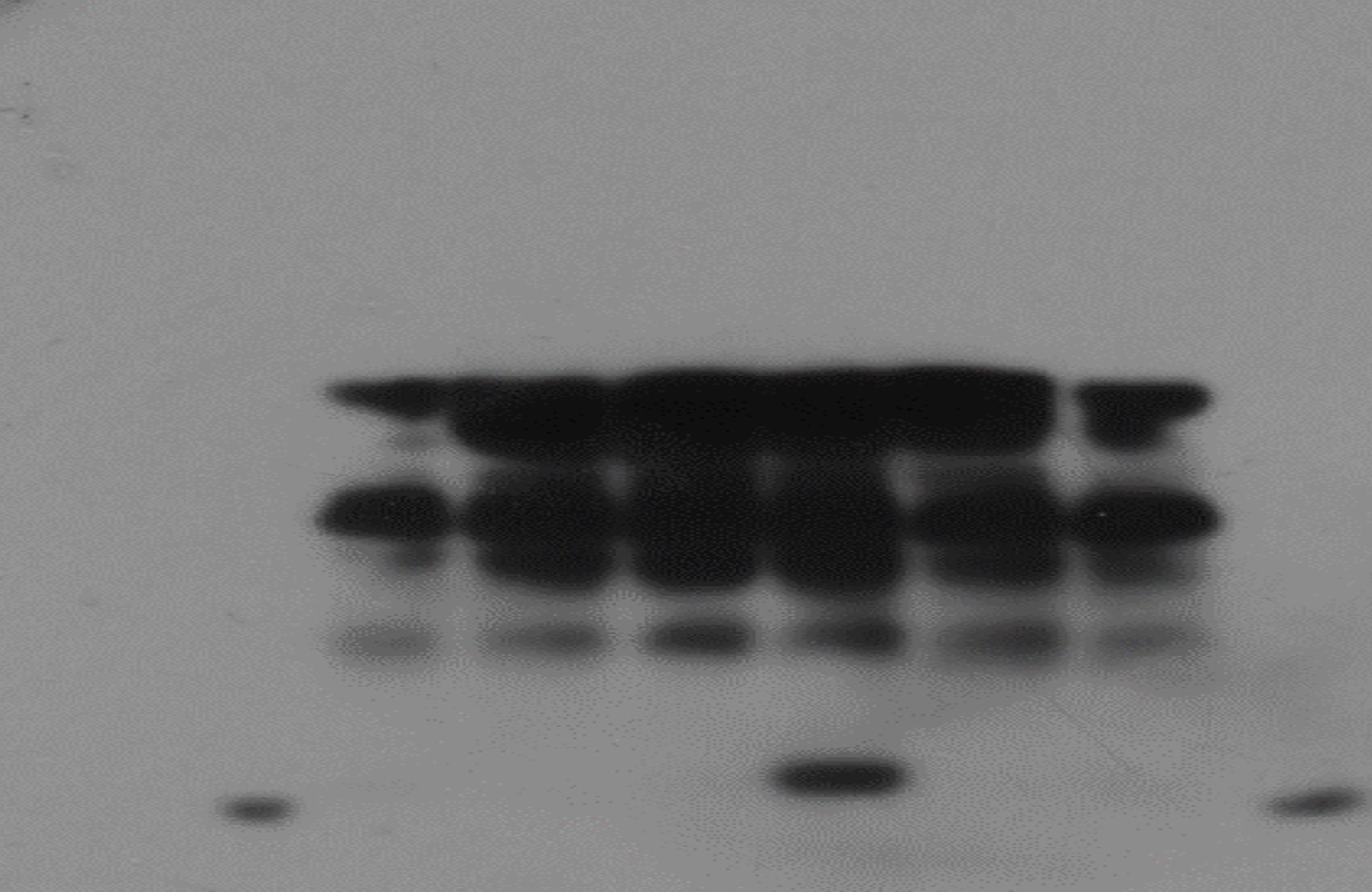

Supplement: Figure 4—source data 3. — Related to Figure 4G. Reduced caspase 1 activation in the presence of Ca2+ chelator BAPTA-AM in monocyte-derived macrophages (MDMs). MDMs were primed with lipopolysaccharide (LPS; 3 hr) and then were pretreated with or without BAPTA-AM (10 µM) for 30 min and subsequently challenged with ATP (5 mM) for 30 min and cell lysates were immunoblotted with anti-Caspase 1. Representative western blotting results from three independent experiments showing reduced caspase 1 activation (reduced Casp-1 p20) when cells were treated with BAPTA-AM. [file elife-83842-fig4-data3.zip › Figure 4 - source data 3/Figure 4 - source data 3-1 for original WB Casp I.jpg]

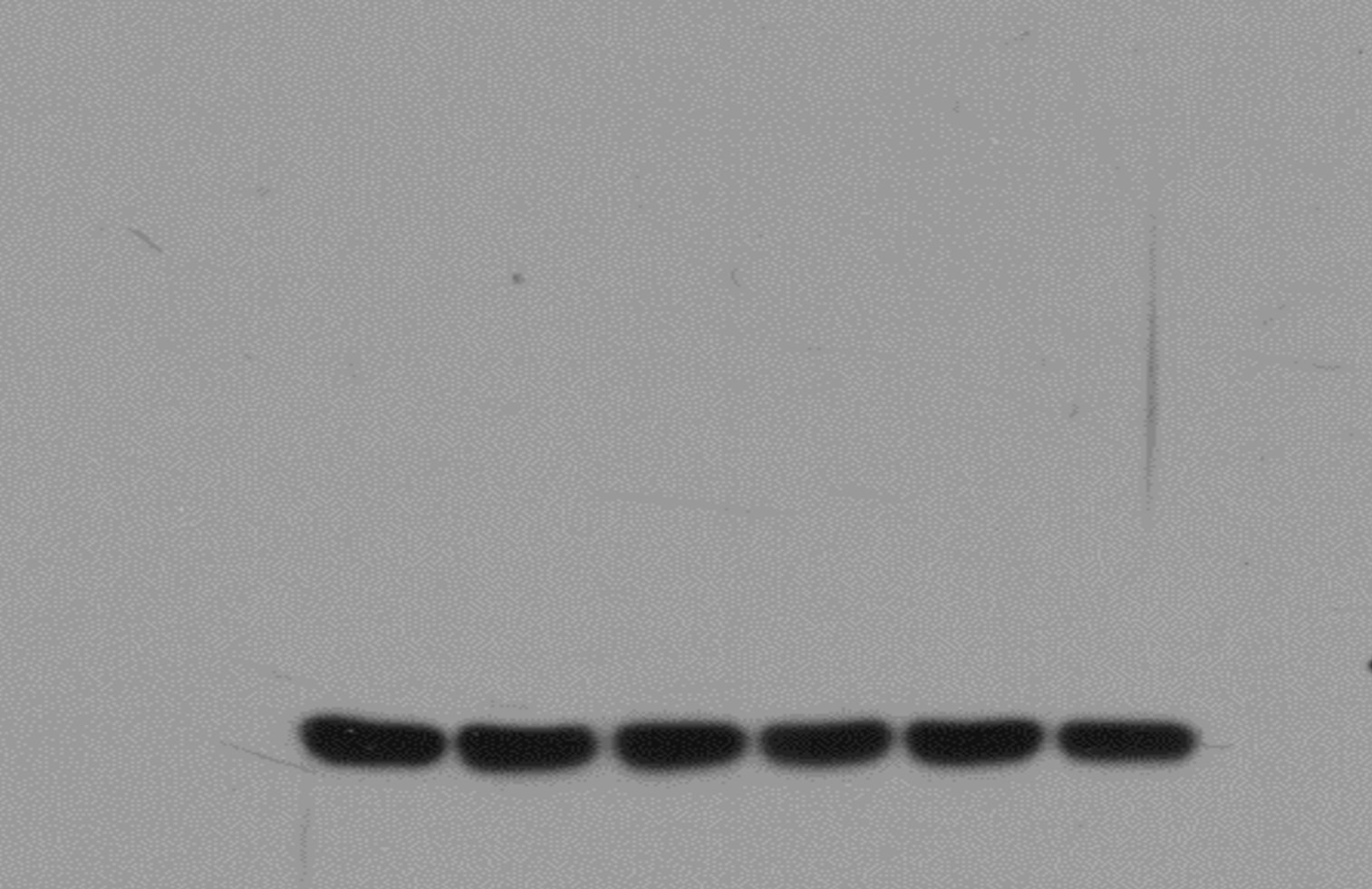

Supplement: Figure 4—source data 3. — Related to Figure 4G. Reduced caspase 1 activation in the presence of Ca2+ chelator BAPTA-AM in monocyte-derived macrophages (MDMs). MDMs were primed with lipopolysaccharide (LPS; 3 hr) and then were pretreated with or without BAPTA-AM (10 µM) for 30 min and subsequently challenged with ATP (5 mM) for 30 min and cell lysates were immunoblotted with anti-Caspase 1. Representative western blotting results from three independent experiments showing reduced caspase 1 activation (reduced Casp-1 p20) when cells were treated with BAPTA-AM. [file elife-83842-fig4-data3.zip › Figure 4 - source data 3/Figure 4 - source data 3-2 for original WB GAPDH.jpg]

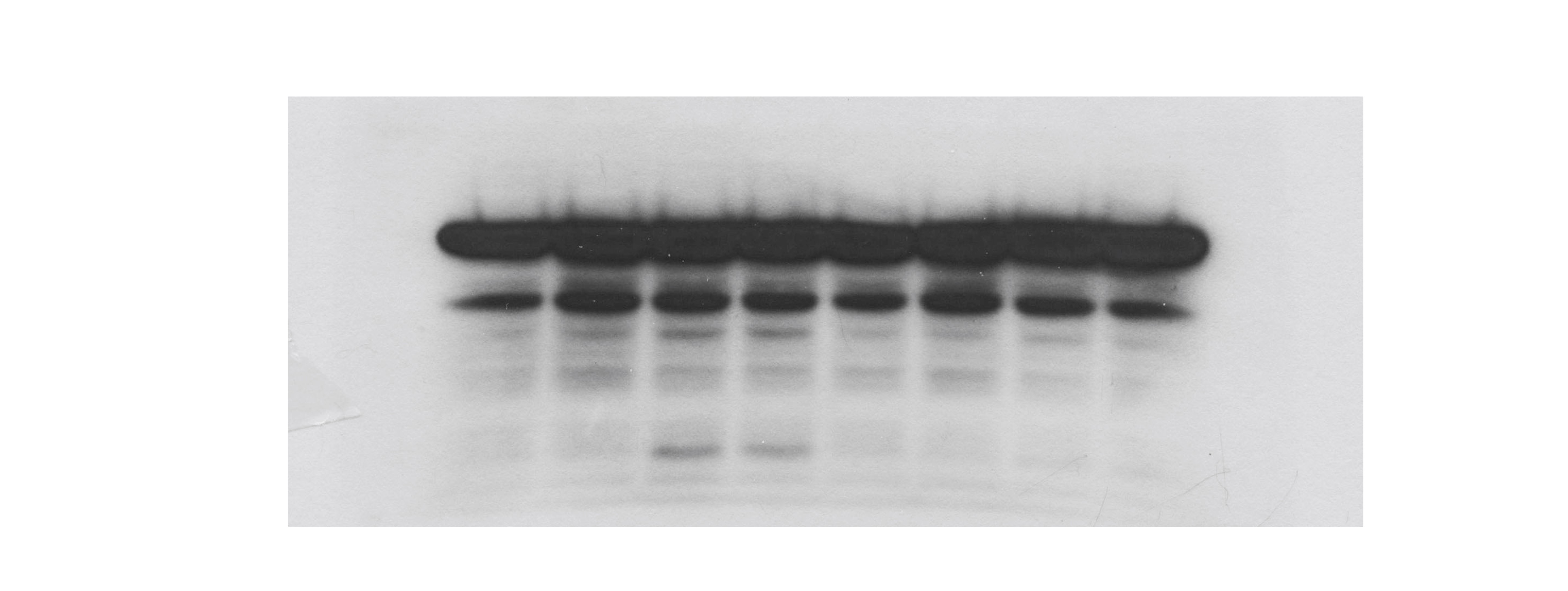

Supplement: Figure 5—source data 1. — Related to Figure 5I. Rab11a-dependent NLRP3 inflammasome activation induced by ATP in macrophages. Inhibited NLRP3 inflammasome activation in monocyte-derived macrophages (MDMs) treated with siRNA targeting mouse Rab11a (siRab11a). Representative results of western blot from three independent experiments showing reduced caspase 1 activation (reduced Casp-1 p20) and IL-1β maturation (reduced IL-1β p17) and Rab11a knocking down after cells were treated with siRab11a in MDMs, but the NLRP3 expression was not affected by siRab11a treatment. MDMs pretreated with siRab11a for 48 hr were primed with lipopolysaccharide (LPS; 3 hr) and subsequently challenged with ATP (5 mM) for 30 min. Cell lysates were immunoblotted with indicated antibodies (anti-TWIK2 or anti-IL1β or anti-Rab11a or anti NLRP3). [file elife-83842-fig5-data1.zip › Figure 5 - source data 1/Figure 5 - source data 1-1 for original WB Casp1.jpg]

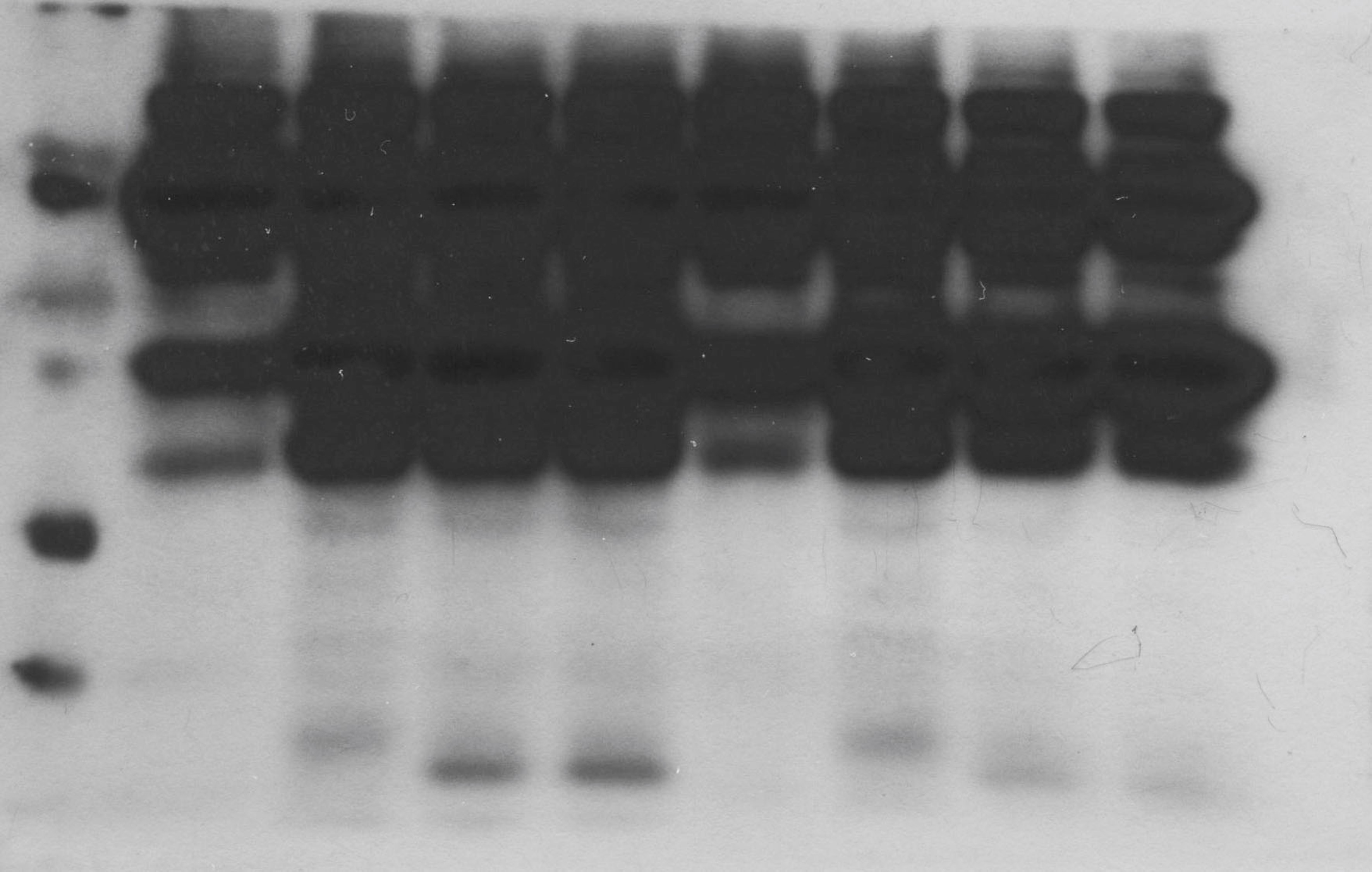

Supplement: Figure 5—source data 1. — Related to Figure 5I. Rab11a-dependent NLRP3 inflammasome activation induced by ATP in macrophages. Inhibited NLRP3 inflammasome activation in monocyte-derived macrophages (MDMs) treated with siRNA targeting mouse Rab11a (siRab11a). Representative results of western blot from three independent experiments showing reduced caspase 1 activation (reduced Casp-1 p20) and IL-1β maturation (reduced IL-1β p17) and Rab11a knocking down after cells were treated with siRab11a in MDMs, but the NLRP3 expression was not affected by siRab11a treatment. MDMs pretreated with siRab11a for 48 hr were primed with lipopolysaccharide (LPS; 3 hr) and subsequently challenged with ATP (5 mM) for 30 min. Cell lysates were immunoblotted with indicated antibodies (anti-TWIK2 or anti-IL1β or anti-Rab11a or anti NLRP3). [file elife-83842-fig5-data1.zip › Figure 5 - source data 1/Figure 5 - source data 1-2 for original WB IL-b.jpg]

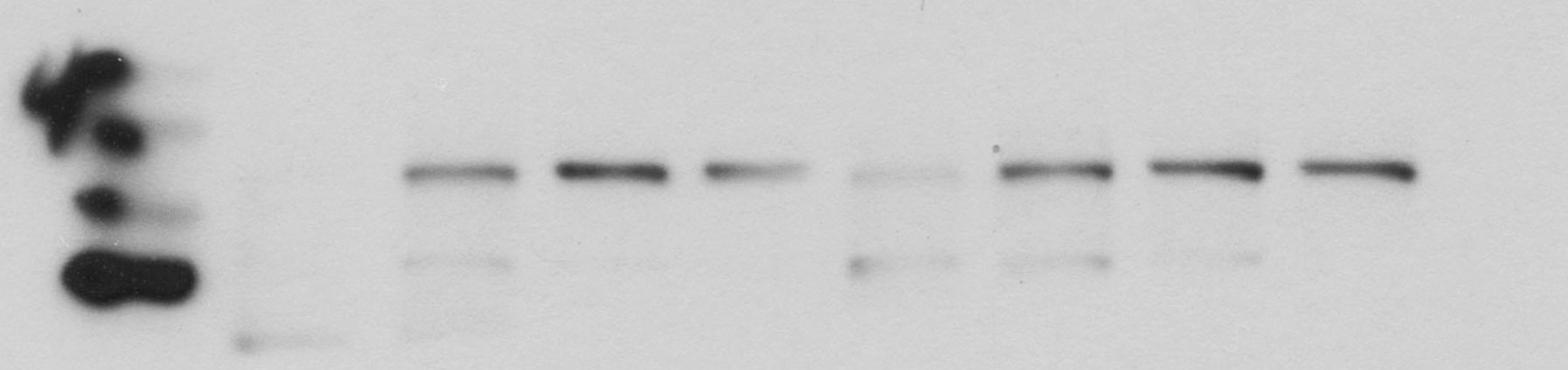

Supplement: Figure 5—source data 1. — Related to Figure 5I. Rab11a-dependent NLRP3 inflammasome activation induced by ATP in macrophages. Inhibited NLRP3 inflammasome activation in monocyte-derived macrophages (MDMs) treated with siRNA targeting mouse Rab11a (siRab11a). Representative results of western blot from three independent experiments showing reduced caspase 1 activation (reduced Casp-1 p20) and IL-1β maturation (reduced IL-1β p17) and Rab11a knocking down after cells were treated with siRab11a in MDMs, but the NLRP3 expression was not affected by siRab11a treatment. MDMs pretreated with siRab11a for 48 hr were primed with lipopolysaccharide (LPS; 3 hr) and subsequently challenged with ATP (5 mM) for 30 min. Cell lysates were immunoblotted with indicated antibodies (anti-TWIK2 or anti-IL1β or anti-Rab11a or anti NLRP3). [file elife-83842-fig5-data1.zip › Figure 5 - source data 1/Figure 5 - source data 1-3 for original WB NLRP3.jpg]

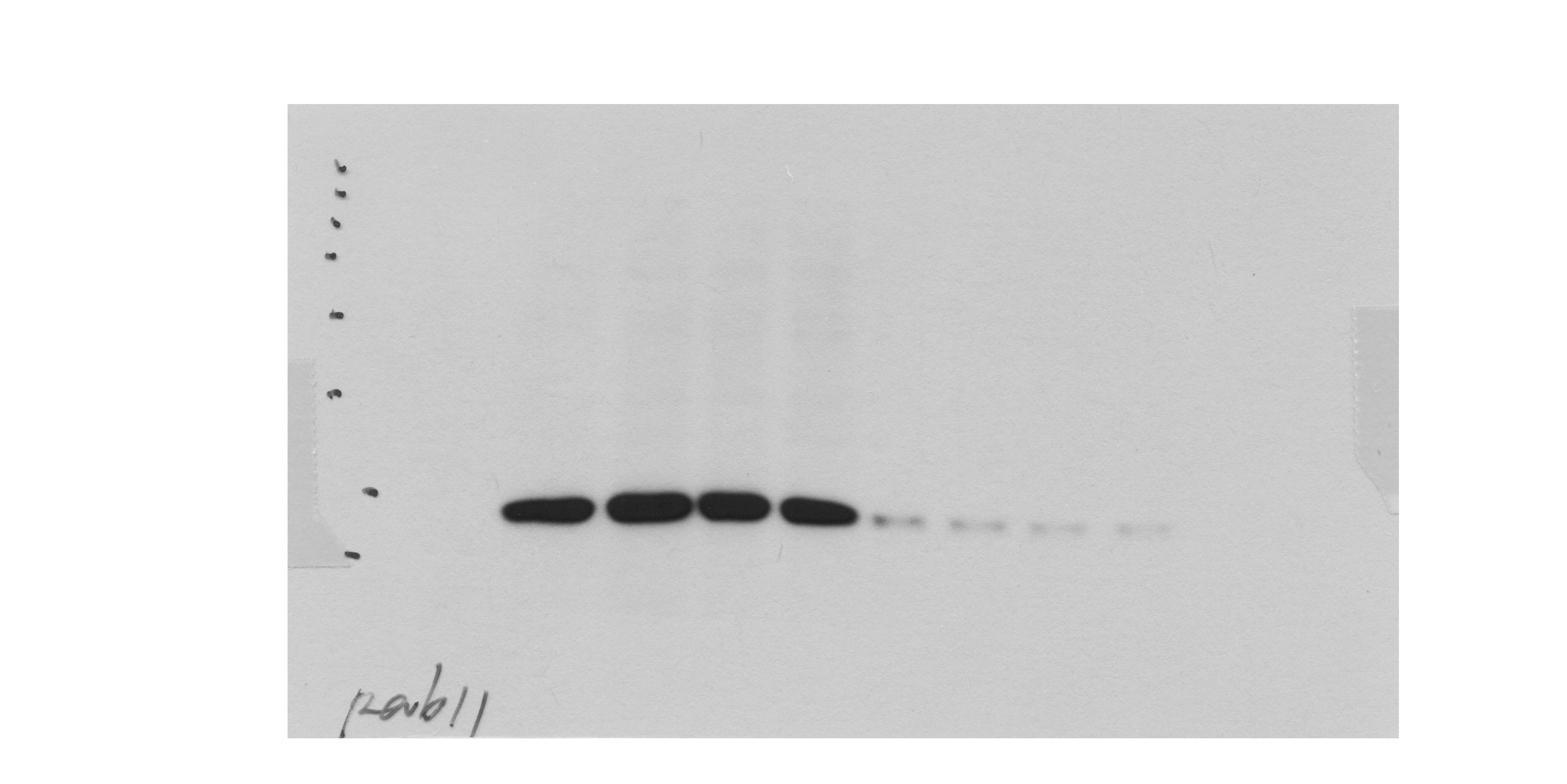

Supplement: Figure 5—source data 1. — Related to Figure 5I. Rab11a-dependent NLRP3 inflammasome activation induced by ATP in macrophages. Inhibited NLRP3 inflammasome activation in monocyte-derived macrophages (MDMs) treated with siRNA targeting mouse Rab11a (siRab11a). Representative results of western blot from three independent experiments showing reduced caspase 1 activation (reduced Casp-1 p20) and IL-1β maturation (reduced IL-1β p17) and Rab11a knocking down after cells were treated with siRab11a in MDMs, but the NLRP3 expression was not affected by siRab11a treatment. MDMs pretreated with siRab11a for 48 hr were primed with lipopolysaccharide (LPS; 3 hr) and subsequently challenged with ATP (5 mM) for 30 min. Cell lysates were immunoblotted with indicated antibodies (anti-TWIK2 or anti-IL1β or anti-Rab11a or anti NLRP3). [file elife-83842-fig5-data1.zip › Figure 5 - source data 1/Figure 5 - source data 1-4 for original WB Rab11a.jpg]

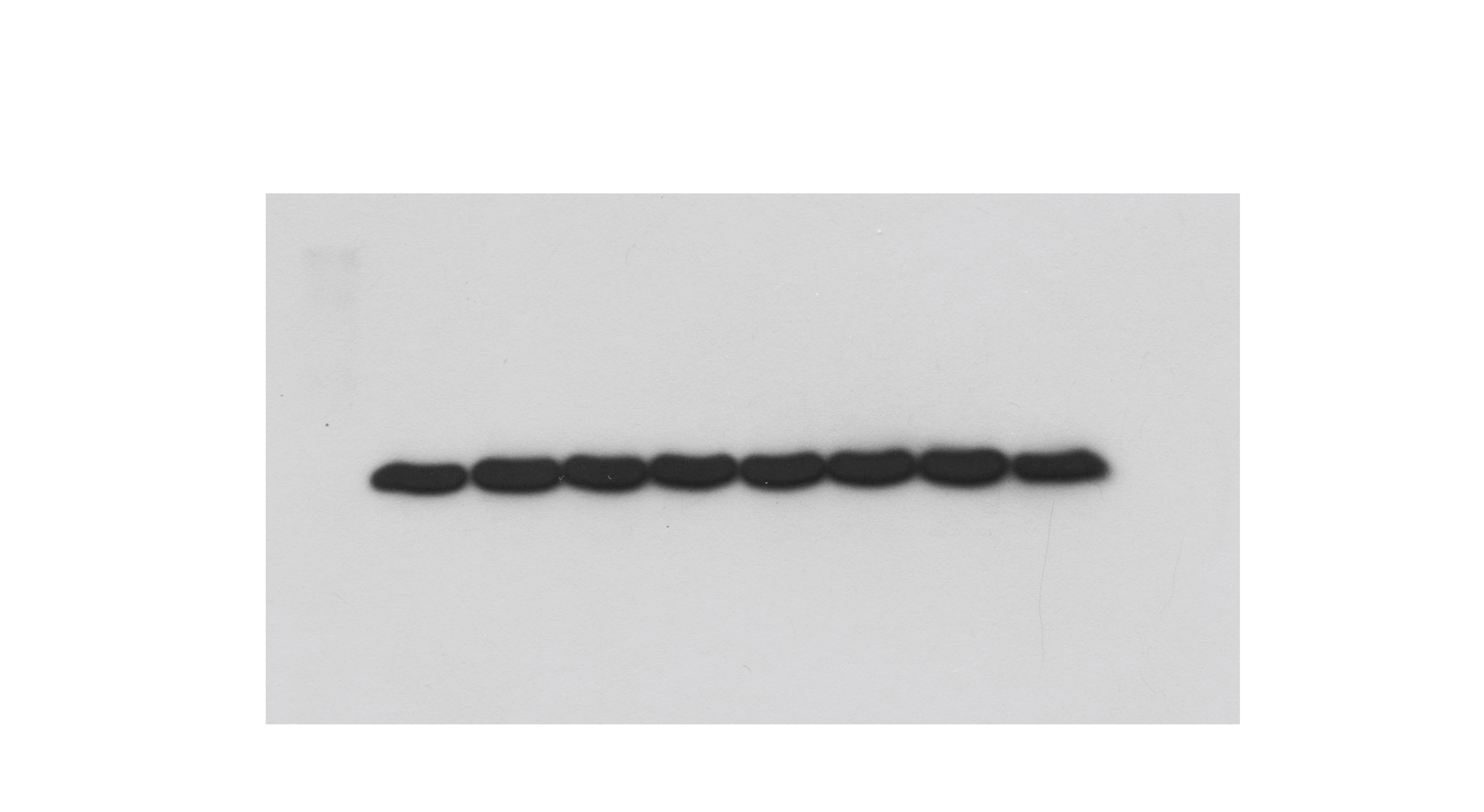

Supplement: Figure 5—source data 1. — Related to Figure 5I. Rab11a-dependent NLRP3 inflammasome activation induced by ATP in macrophages. Inhibited NLRP3 inflammasome activation in monocyte-derived macrophages (MDMs) treated with siRNA targeting mouse Rab11a (siRab11a). Representative results of western blot from three independent experiments showing reduced caspase 1 activation (reduced Casp-1 p20) and IL-1β maturation (reduced IL-1β p17) and Rab11a knocking down after cells were treated with siRab11a in MDMs, but the NLRP3 expression was not affected by siRab11a treatment. MDMs pretreated with siRab11a for 48 hr were primed with lipopolysaccharide (LPS; 3 hr) and subsequently challenged with ATP (5 mM) for 30 min. Cell lysates were immunoblotted with indicated antibodies (anti-TWIK2 or anti-IL1β or anti-Rab11a or anti NLRP3). [file elife-83842-fig5-data1.zip › Figure 5 - source data 1/Figure 5 - source data 1-5 for original WB GAPDH.jpg]

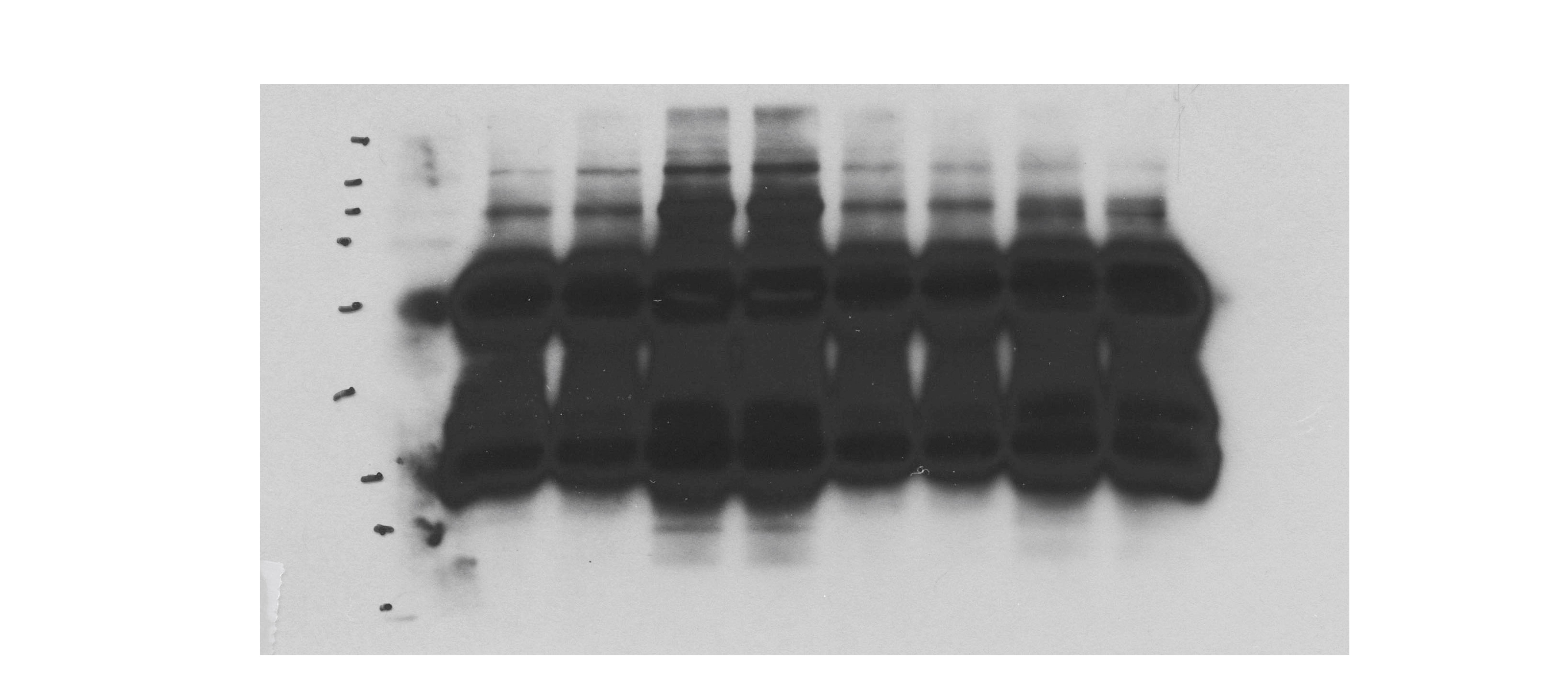

Supplement: Figure 6—source data 1. — Related to Figure 6B. Lung macrophages (Mac) were depleted with clodronate liposomes and then reconstituted via intratracheal route with monocyte-derived macrophages (MDMs) treated with either siRNA of Rab11a or siRNA control as illustrated. The mice were injected with lipopolysaccharide (LPS; intra-peritoneal injection, i.p.) after 24 hr of macrophage reconstitution. Lungs were harvested for evaluation of NLRP3 inflammasome activation and lung inflammation. NLRP3 inflammasome activation (indicated by caspase 1 activation and IL-1β maturation) in the murine lung was assessed by immunoblotting. [file elife-83842-fig6-data1.zip › Figure 6 - source data 1/Figure 6B-source data 1 for original WB Casp 1.jpg]

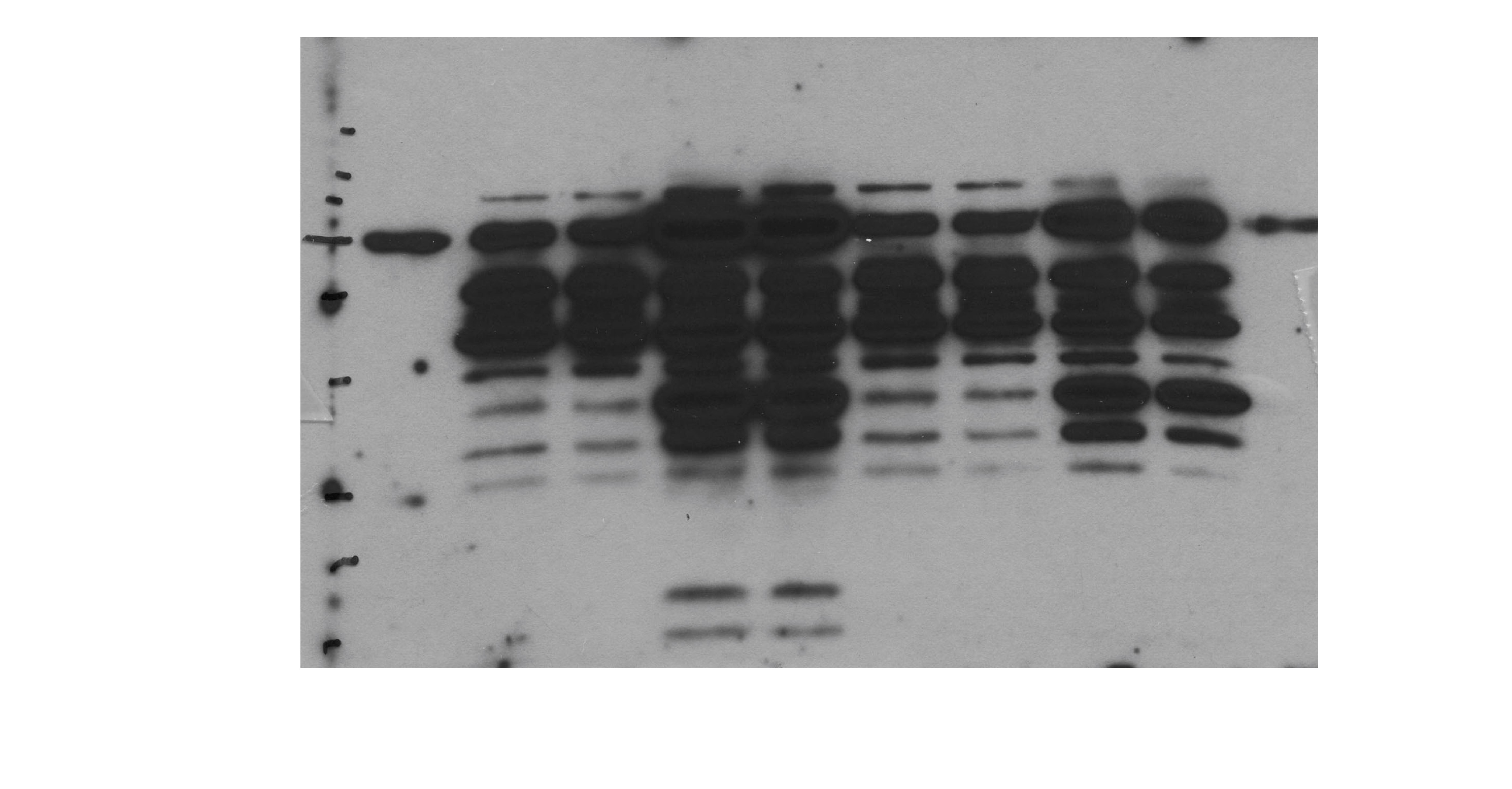

Supplement: Figure 6—source data 1. — Related to Figure 6B. Lung macrophages (Mac) were depleted with clodronate liposomes and then reconstituted via intratracheal route with monocyte-derived macrophages (MDMs) treated with either siRNA of Rab11a or siRNA control as illustrated. The mice were injected with lipopolysaccharide (LPS; intra-peritoneal injection, i.p.) after 24 hr of macrophage reconstitution. Lungs were harvested for evaluation of NLRP3 inflammasome activation and lung inflammation. NLRP3 inflammasome activation (indicated by caspase 1 activation and IL-1β maturation) in the murine lung was assessed by immunoblotting. [file elife-83842-fig6-data1.zip › Figure 6 - source data 1/Figure 6B-source data 2 for original WB IL-1b.jpg]

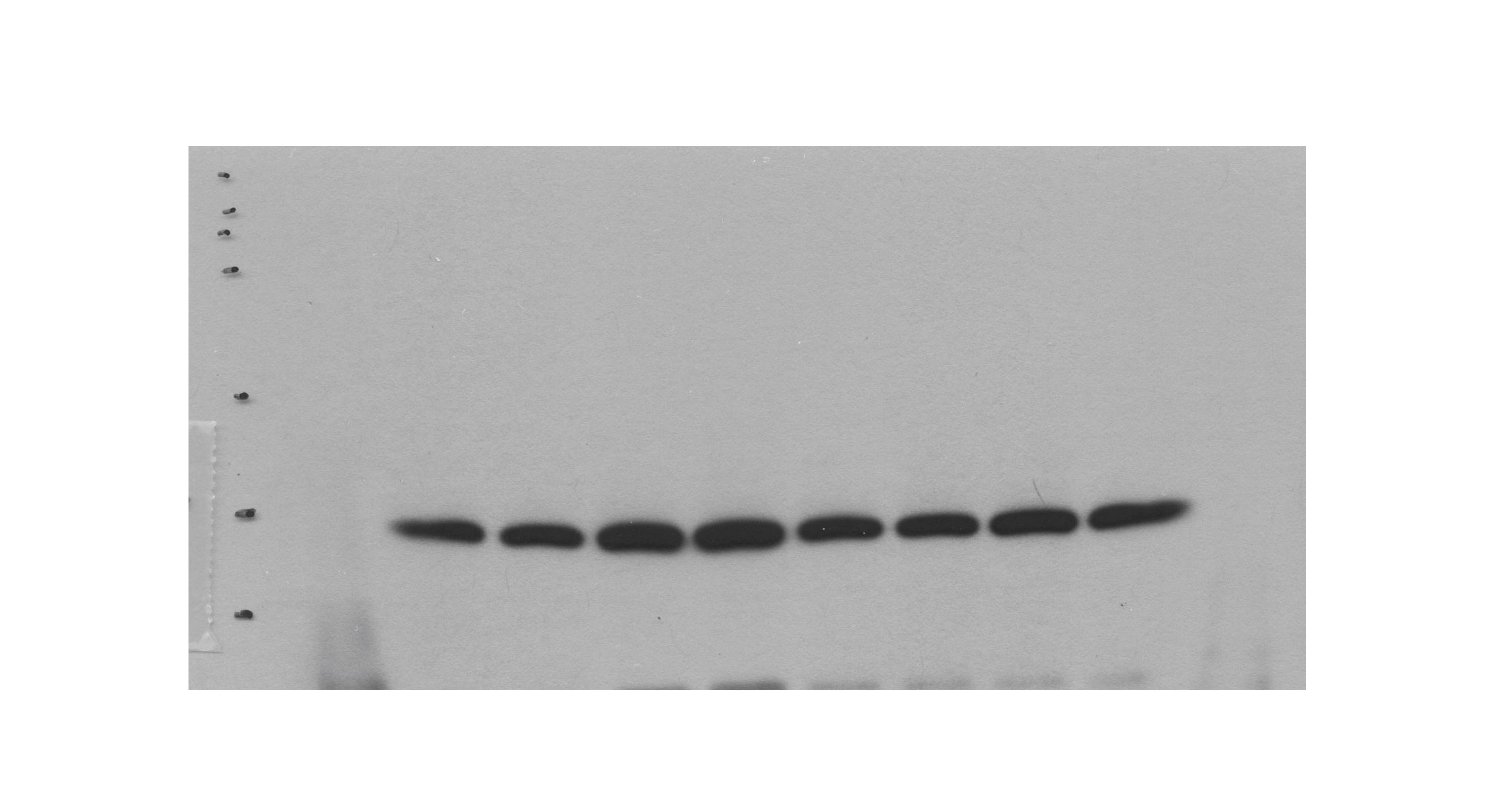

Supplement: Figure 6—source data 1. — Related to Figure 6B. Lung macrophages (Mac) were depleted with clodronate liposomes and then reconstituted via intratracheal route with monocyte-derived macrophages (MDMs) treated with either siRNA of Rab11a or siRNA control as illustrated. The mice were injected with lipopolysaccharide (LPS; intra-peritoneal injection, i.p.) after 24 hr of macrophage reconstitution. Lungs were harvested for evaluation of NLRP3 inflammasome activation and lung inflammation. NLRP3 inflammasome activation (indicated by caspase 1 activation and IL-1β maturation) in the murine lung was assessed by immunoblotting. [file elife-83842-fig6-data1.zip › Figure 6 - source data 1/Figure 6B-source data 3 for original WB GAPDH.jpg]
