# Supplementary material for: Endosomal trafficking of two-pore K+ efflux channel TWIK2 to plasmalemma mediates NLRP3 inflammasome activation and inflammatory injury
Source: eLife. 2023 May 9;12:e83842. doi: 10.7554/eLife.83842 (PMC10202452; doi:10.7554/eLife.83842)
Supplement: Figure 3—source data 2. — Related to Figure 3E. Representative results of western blot from three independent experiments showing reduced IL-1β maturation (reduced IL-1β p17). Monocyte-derived macrophages (MDMs) pretreated with vesicle–plasmalemma fusion inhibitor Vacuolin (10 µM, 2 hr) were primed with lipopolysaccharide (LPS; 3 hr) and subsequently challenged with ATP (5 mM) for 30 min. Cell lysates or pellets were immunoblotted with indicated antibodies (anti-TWIK2 or anti-IL1β). [file elife-83842-fig3-data2.zip › Figure 3 - source data 2/Figure 3 - source data 2 for WB labelled.pptx]

## Slide 1
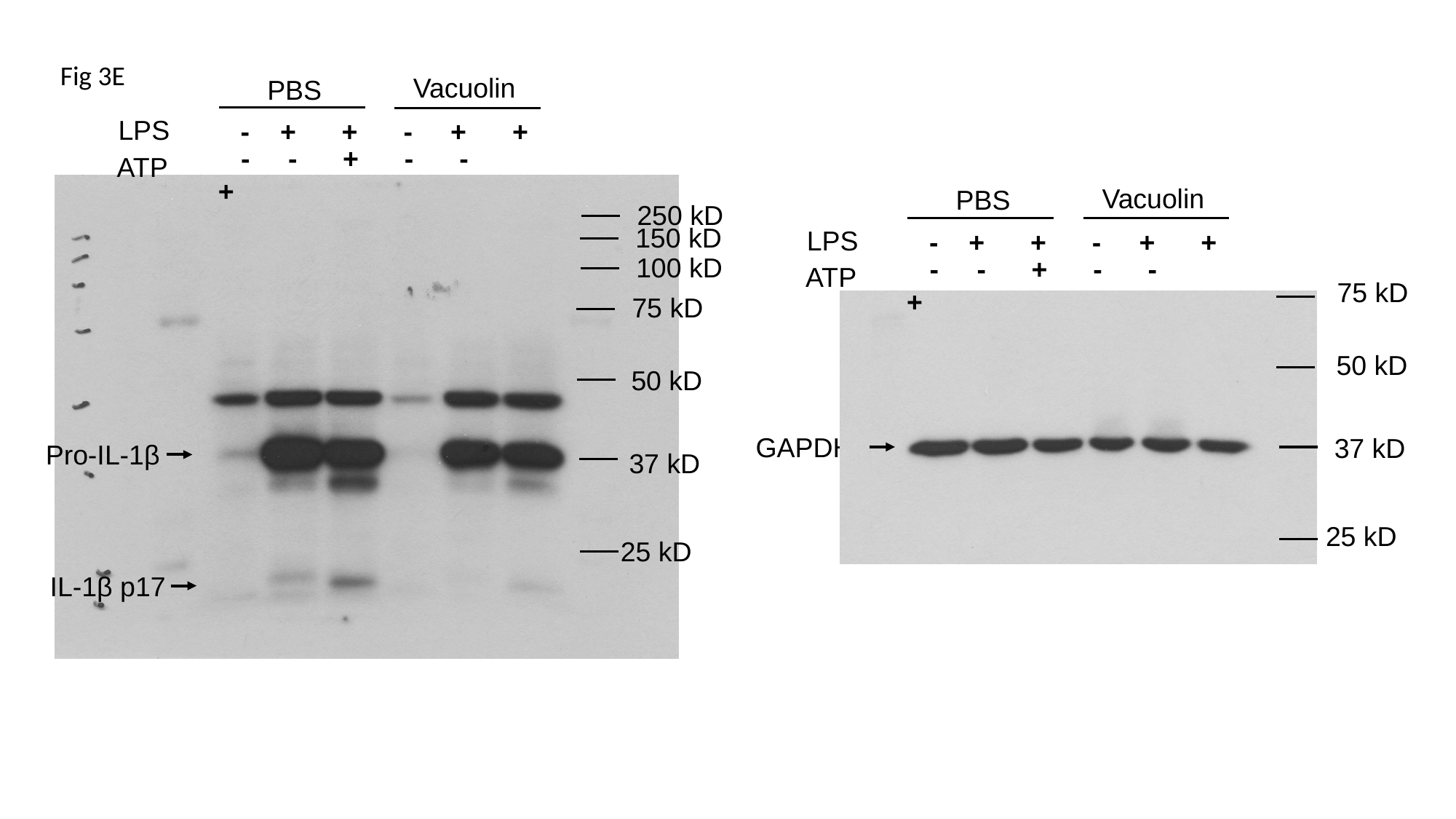

Fig 3E
Vacuolin
PBS
LPS
 - + + - + +
 - - + - - +
ATP
Vacuolin
PBS
250 kD
150 kD
LPS
 - + + - + +
100 kD
 - - + - - +
ATP
75 kD
75 kD
50 kD
50 kD
GAPDH
37 kD
Pro-IL-1β
37 kD
25 kD
25 kD
IL-1β p17
