# Supplementary material for: Endosomal trafficking of two-pore K+ efflux channel TWIK2 to plasmalemma mediates NLRP3 inflammasome activation and inflammatory injury
Source: eLife. 2023 May 9;12:e83842. doi: 10.7554/eLife.83842 (PMC10202452; doi:10.7554/eLife.83842)
Supplement: Figure 4—source data 1. — Related to Figure 4C. Extracellular Ca2+-dependent NLRP3 inflammasome activation in macrophages. Monocyte-derived macrophages (MDMs) were primed with lipopolysaccharide (LPS) and subsequently challenged with ATP and cell lysates or pellets were immunoblotted with indicated antibodies (anti-Caspase 1 or anti-IL1β). Representative western blotting results from three independent experiments showing reduced caspase 1 activation (reduced Casp-1 p20) in the absence of extracellular Ca2+. [file elife-83842-fig4-data1.zip › Figure 4 - source data 1/Figure 4.pptx]

## Slide 1
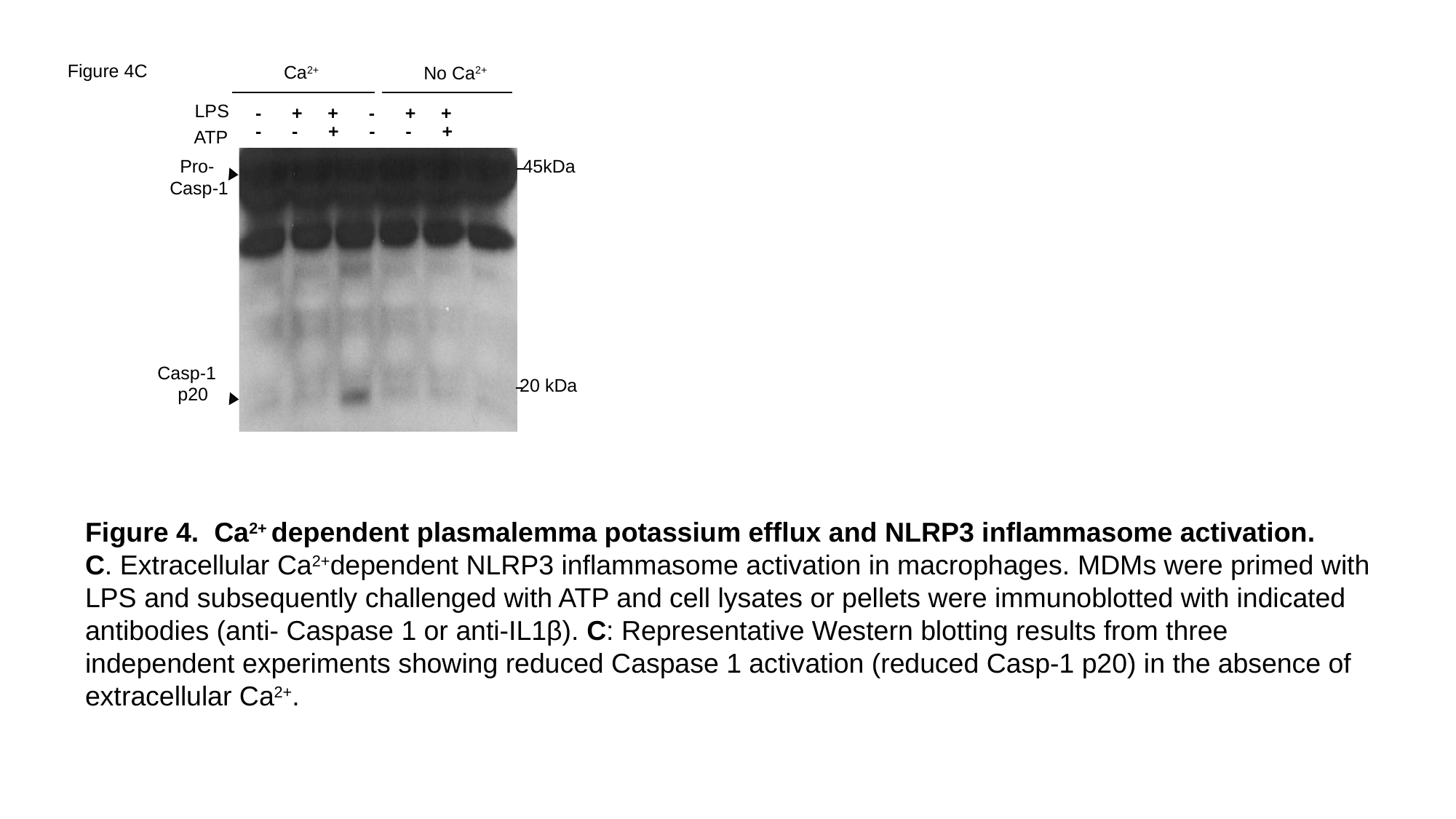

Figure 4C
Ca2+
No Ca2+
LPS
- + + - + +
- - + - - +
ATP
45kDa
 Pro-
Casp-1
Casp-1
 p20
20 kDa
Figure 4. Ca2+ dependent plasmalemma potassium efflux and NLRP3 inflammasome activation.
C. Extracellular Ca2+dependent NLRP3 inflammasome activation in macrophages. MDMs were primed with LPS and subsequently challenged with ATP and cell lysates or pellets were immunoblotted with indicated antibodies (anti- Caspase 1 or anti-IL1β). C: Representative Western blotting results from three independent experiments showing reduced Caspase 1 activation (reduced Casp-1 p20) in the absence of extracellular Ca2+.
