# Supplementary material for: Endosomal trafficking of two-pore K+ efflux channel TWIK2 to plasmalemma mediates NLRP3 inflammasome activation and inflammatory injury
Source: eLife. 2023 May 9;12:e83842. doi: 10.7554/eLife.83842 (PMC10202452; doi:10.7554/eLife.83842)
Supplement: Figure 4—source data 3. — Related to Figure 4G. Reduced caspase 1 activation in the presence of Ca2+ chelator BAPTA-AM in monocyte-derived macrophages (MDMs). MDMs were primed with lipopolysaccharide (LPS; 3 hr) and then were pretreated with or without BAPTA-AM (10 µM) for 30 min and subsequently challenged with ATP (5 mM) for 30 min and cell lysates were immunoblotted with anti-Caspase 1. Representative western blotting results from three independent experiments showing reduced caspase 1 activation (reduced Casp-1 p20) when cells were treated with BAPTA-AM. [file elife-83842-fig4-data3.zip › Figure 4 - source data 3/Figure 4.pptx]

## Slide 1
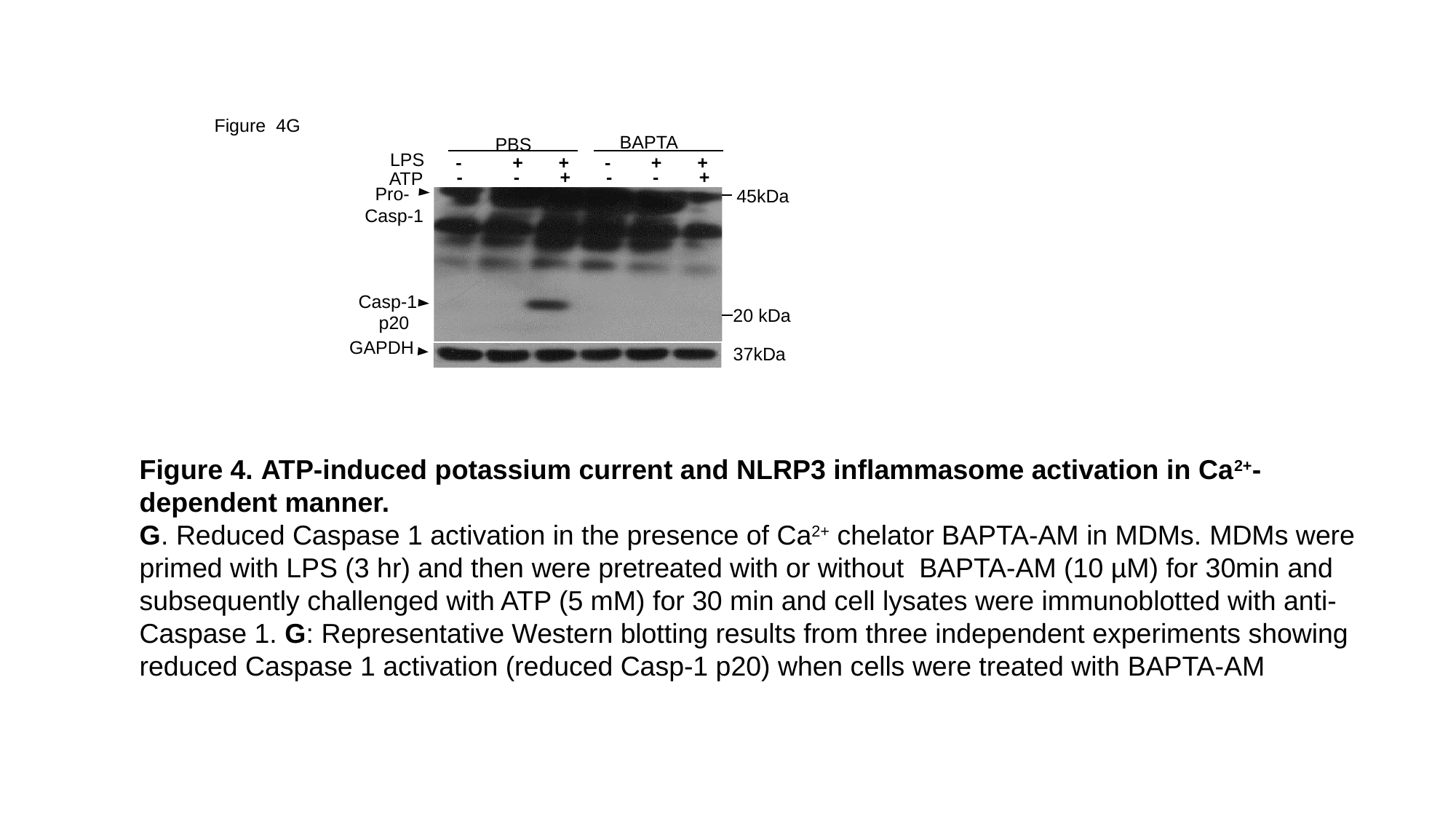

Figure 4G
 BAPTA
PBS
 - - + - - +
ATP
 Pro-
Casp-1
45kDa
Casp-1
 p20
20 kDa
LPS
 - + + - + +
GAPDH
37kDa
Figure 4. ATP-induced potassium current and NLRP3 inflammasome activation in Ca2+- dependent manner.
G. Reduced Caspase 1 activation in the presence of Ca2+ chelator BAPTA-AM in MDMs. MDMs were primed with LPS (3 hr) and then were pretreated with or without BAPTA-AM (10 µM) for 30min and subsequently challenged with ATP (5 mM) for 30 min and cell lysates were immunoblotted with anti-Caspase 1. G: Representative Western blotting results from three independent experiments showing reduced Caspase 1 activation (reduced Casp-1 p20) when cells were treated with BAPTA-AM
