# Supplementary material for: Endosomal trafficking of two-pore K+ efflux channel TWIK2 to plasmalemma mediates NLRP3 inflammasome activation and inflammatory injury
Source: eLife. 2023 May 9;12:e83842. doi: 10.7554/eLife.83842 (PMC10202452; doi:10.7554/eLife.83842)
Supplement: Figure 5—source data 1. — Related to Figure 5I. Rab11a-dependent NLRP3 inflammasome activation induced by ATP in macrophages. Inhibited NLRP3 inflammasome activation in monocyte-derived macrophages (MDMs) treated with siRNA targeting mouse Rab11a (siRab11a). Representative results of western blot from three independent experiments showing reduced caspase 1 activation (reduced Casp-1 p20) and IL-1β maturation (reduced IL-1β p17) and Rab11a knocking down after cells were treated with siRab11a in MDMs, but the NLRP3 expression was not affected by siRab11a treatment. MDMs pretreated with siRab11a for 48 hr were primed with lipopolysaccharide (LPS; 3 hr) and subsequently challenged with ATP (5 mM) for 30 min. Cell lysates were immunoblotted with indicated antibodies (anti-TWIK2 or anti-IL1β or anti-Rab11a or anti NLRP3). [file elife-83842-fig5-data1.zip › Figure 5 - source data 1/Figure 5 - Source data 1 for WB labelled.pptx]

## Slide 1
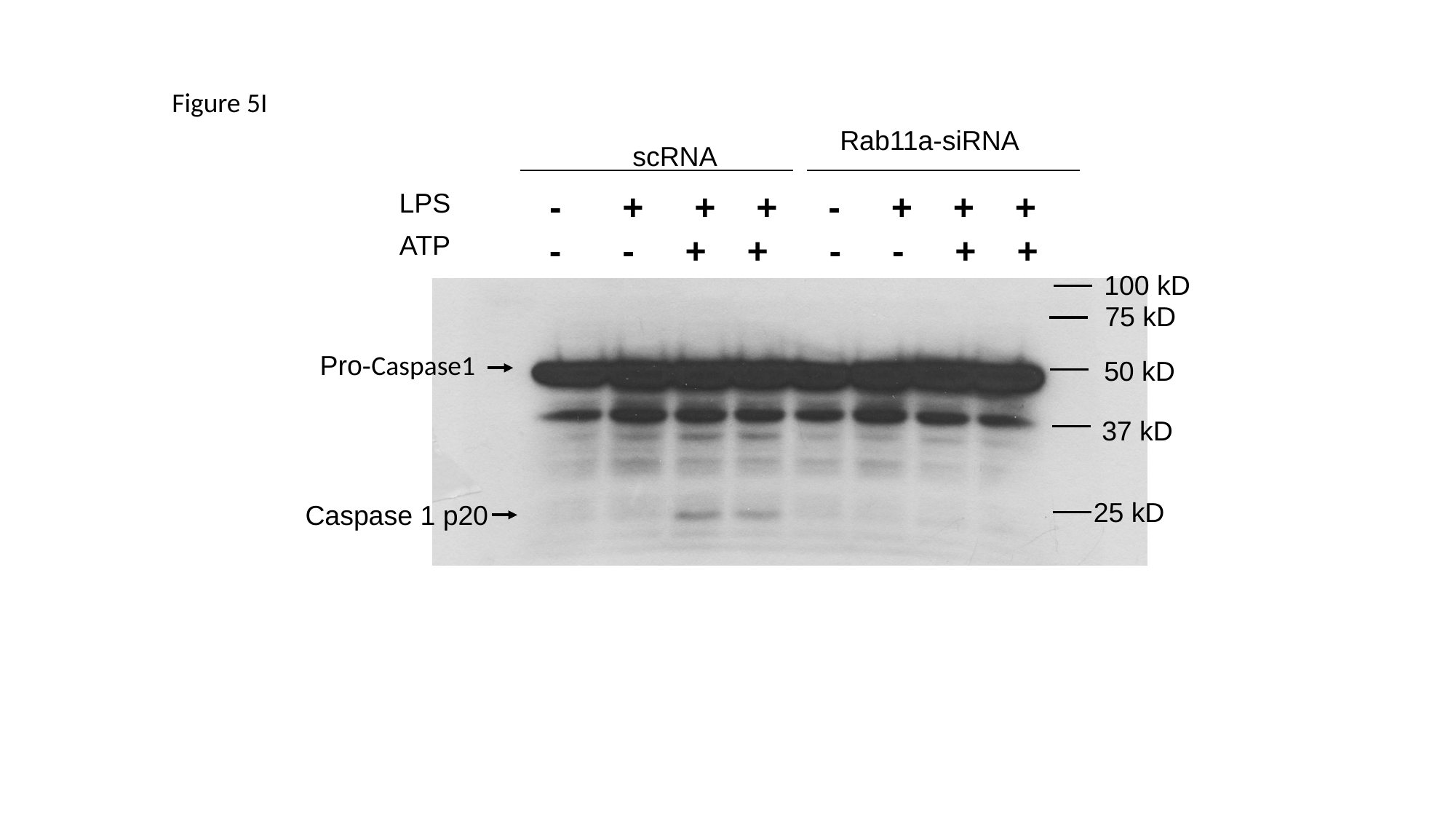

Figure 5I
Rab11a-siRNA
scRNA
- + + + - + + +
LPS
- - + + - - + +
ATP
100 kD
75 kD
Pro-Caspase1
50 kD
37 kD
25 kD
 Caspase 1 p20

## Slide 2
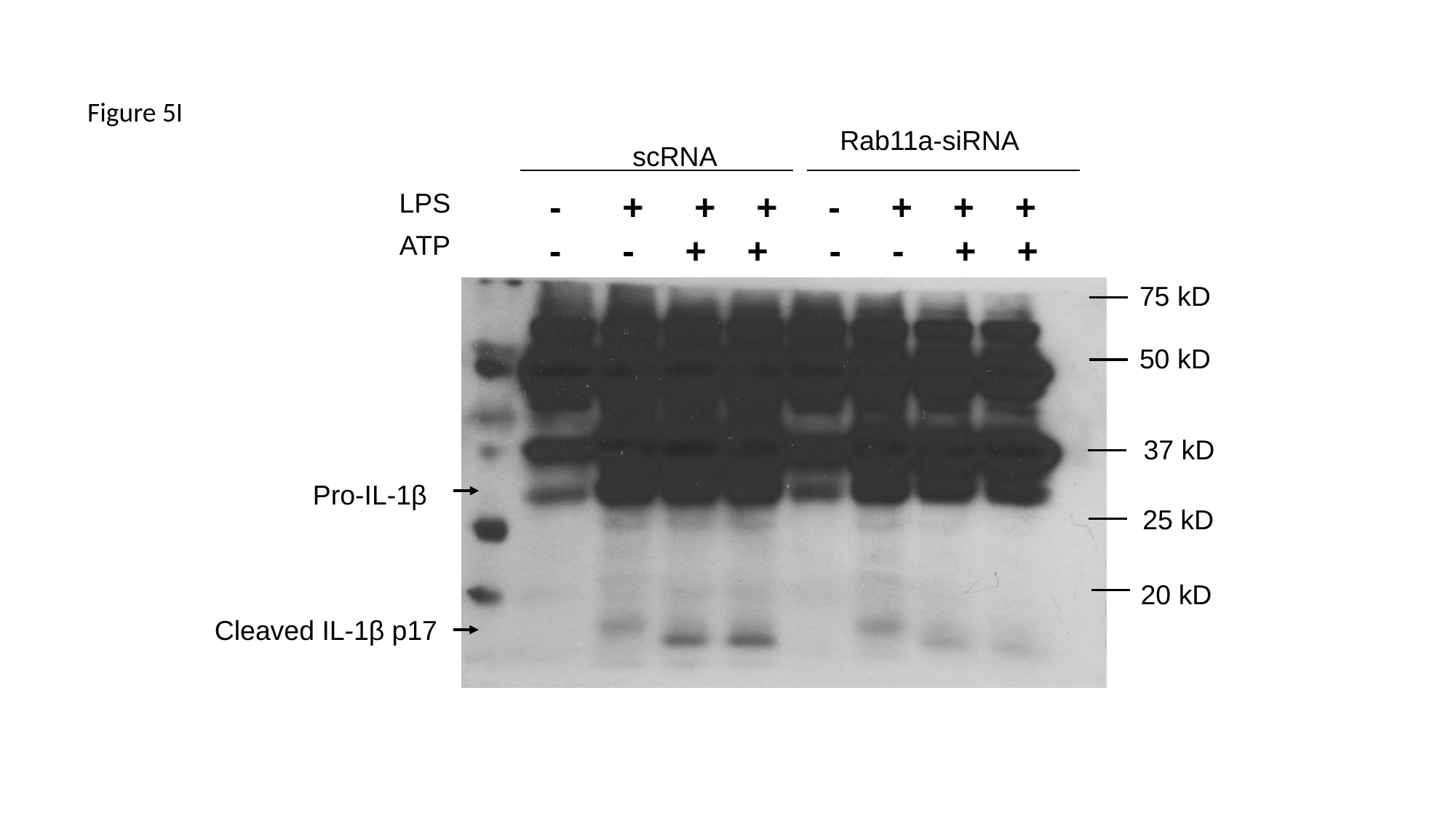

Figure 5I
Rab11a-siRNA
scRNA
- + + + - + + +
LPS
- - + + - - + +
ATP
75 kD
50 kD
37 kD
Pro-IL-1β
25 kD
20 kD
 Cleaved IL-1β p17

## Slide 3
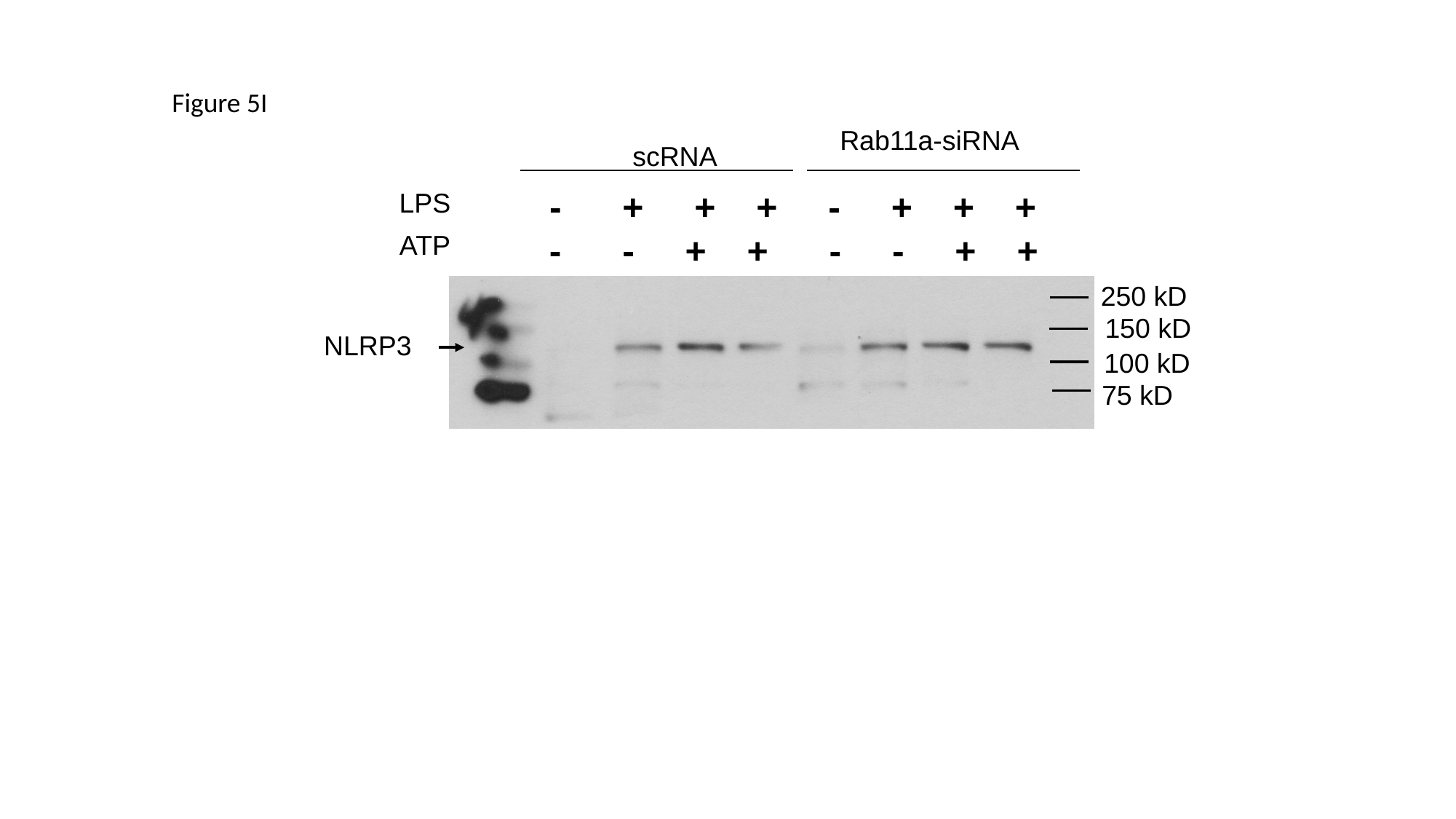

Figure 5I
Rab11a-siRNA
scRNA
- + + + - + + +
LPS
- - + + - - + +
ATP
250 kD
150 kD
NLRP3
100 kD
75 kD

## Slide 4
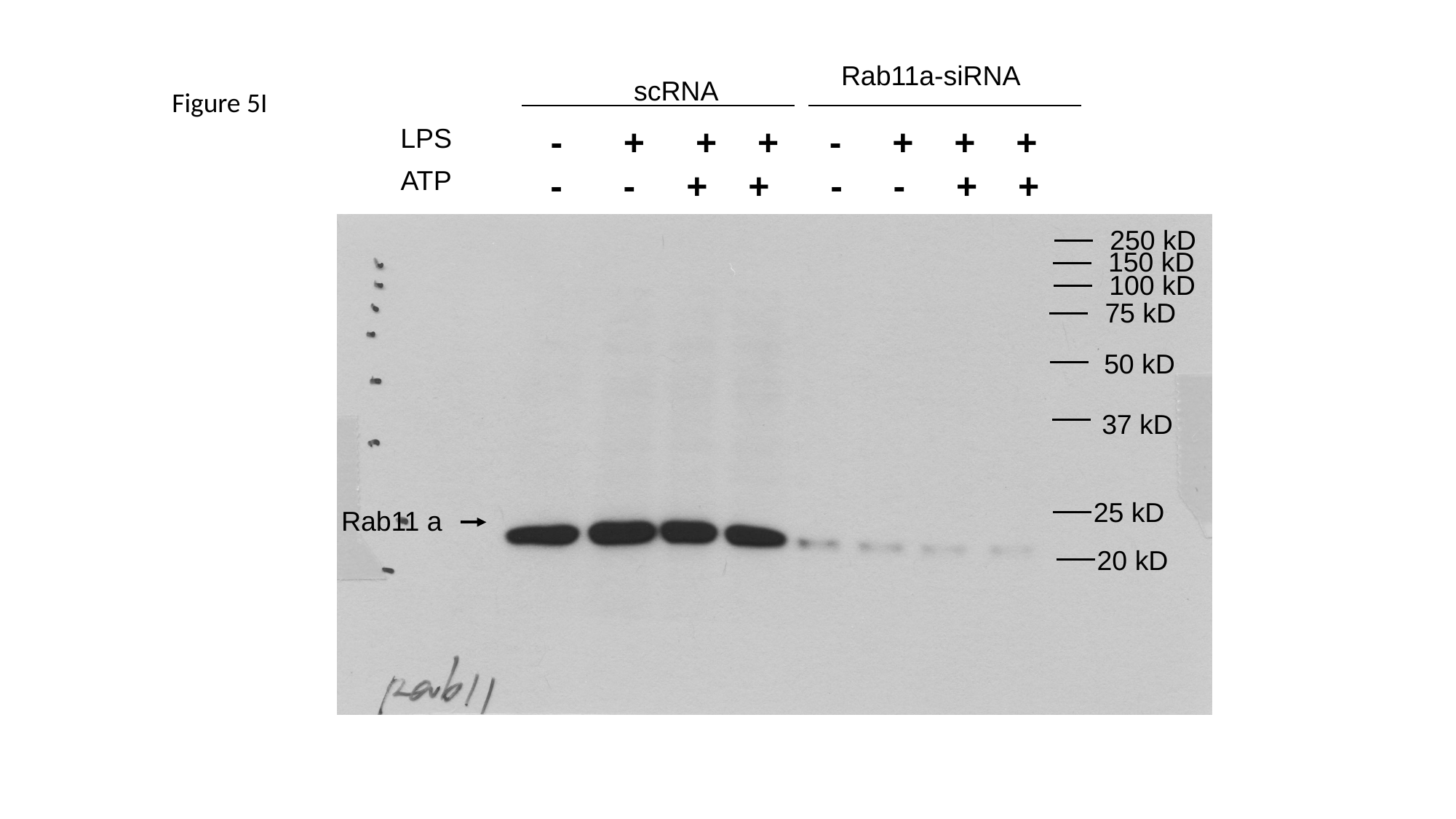

Rab11a-siRNA
scRNA
Figure 5I
- + + + - + + +
LPS
- - + + - - + +
ATP
250 kD
150 kD
100 kD
75 kD
50 kD
37 kD
25 kD
Rab11 a
20 kD

## Slide 5
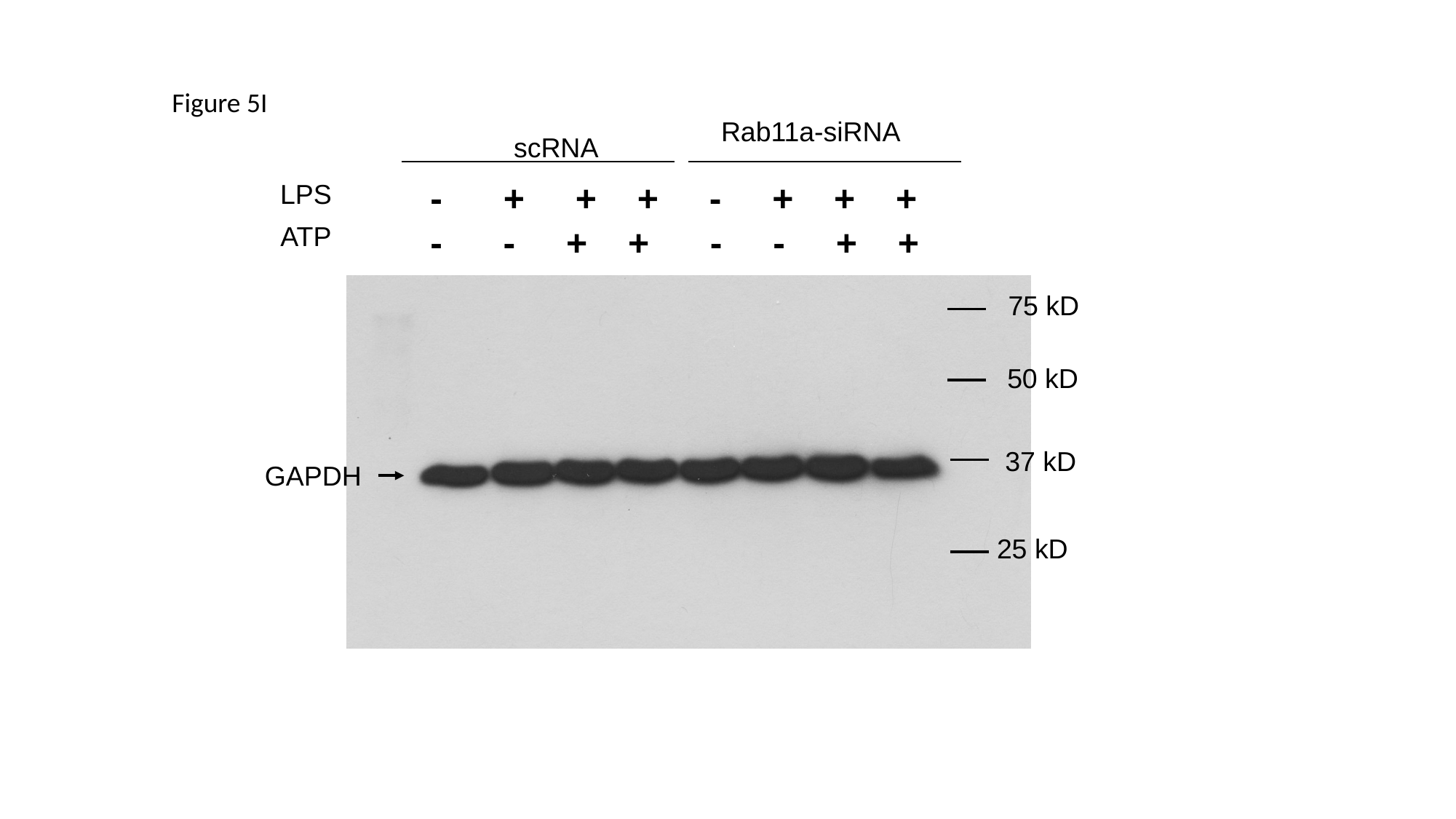

Figure 5I
Rab11a-siRNA
scRNA
- + + + - + + +
LPS
- - + + - - + +
ATP
75 kD
50 kD
37 kD
GAPDH
25 kD
