# Supplementary material for: Endosomal trafficking of two-pore K+ efflux channel TWIK2 to plasmalemma mediates NLRP3 inflammasome activation and inflammatory injury
Source: eLife. 2023 May 9;12:e83842. doi: 10.7554/eLife.83842 (PMC10202452; doi:10.7554/eLife.83842)
Supplement: Figure 5—source data 1. — Related to Figure 5I. Rab11a-dependent NLRP3 inflammasome activation induced by ATP in macrophages. Inhibited NLRP3 inflammasome activation in monocyte-derived macrophages (MDMs) treated with siRNA targeting mouse Rab11a (siRab11a). Representative results of western blot from three independent experiments showing reduced caspase 1 activation (reduced Casp-1 p20) and IL-1β maturation (reduced IL-1β p17) and Rab11a knocking down after cells were treated with siRab11a in MDMs, but the NLRP3 expression was not affected by siRab11a treatment. MDMs pretreated with siRab11a for 48 hr were primed with lipopolysaccharide (LPS; 3 hr) and subsequently challenged with ATP (5 mM) for 30 min. Cell lysates were immunoblotted with indicated antibodies (anti-TWIK2 or anti-IL1β or anti-Rab11a or anti NLRP3). [file elife-83842-fig5-data1.zip › Figure 5 - source data 1/Figure 5.pptx]

## Slide 1
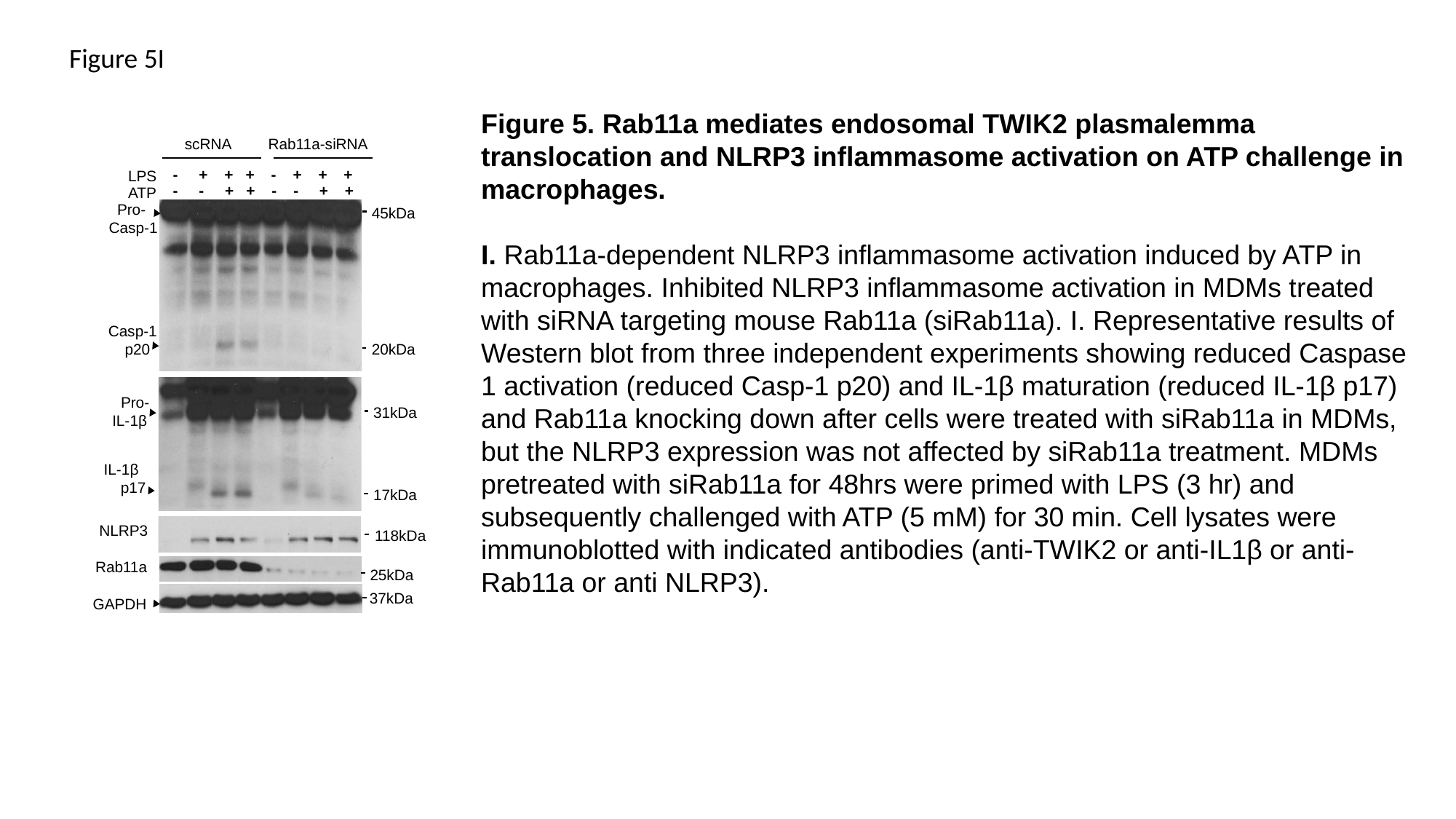

Figure 5I
Figure 5. Rab11a mediates endosomal TWIK2 plasmalemma translocation and NLRP3 inflammasome activation on ATP challenge in macrophages.
I. Rab11a-dependent NLRP3 inflammasome activation induced by ATP in macrophages. Inhibited NLRP3 inflammasome activation in MDMs treated with siRNA targeting mouse Rab11a (siRab11a). I. Representative results of Western blot from three independent experiments showing reduced Caspase 1 activation (reduced Casp-1 p20) and IL-1β maturation (reduced IL-1β p17) and Rab11a knocking down after cells were treated with siRab11a in MDMs, but the NLRP3 expression was not affected by siRab11a treatment. MDMs pretreated with siRab11a for 48hrs were primed with LPS (3 hr) and subsequently challenged with ATP (5 mM) for 30 min. Cell lysates were immunoblotted with indicated antibodies (anti-TWIK2 or anti-IL1β or anti-Rab11a or anti NLRP3).
 scRNA
Rab11a-siRNA
 - + + + - + + +
LPS
 - - + + - - + +
ATP
 Pro-
Casp-1
45kDa
Casp-1
 p20
20kDa
 Pro-
IL-1β
31kDa
IL-1β
 p17
17kDa
NLRP3
118kDa
Rab11a
25kDa
37kDa
GAPDH
