# Supplementary material for: Endosomal trafficking of two-pore K+ efflux channel TWIK2 to plasmalemma mediates NLRP3 inflammasome activation and inflammatory injury
Source: eLife. 2023 May 9;12:e83842. doi: 10.7554/eLife.83842 (PMC10202452; doi:10.7554/eLife.83842)
Supplement: Figure 6—source data 1. — Related to Figure 6B. Lung macrophages (Mac) were depleted with clodronate liposomes and then reconstituted via intratracheal route with monocyte-derived macrophages (MDMs) treated with either siRNA of Rab11a or siRNA control as illustrated. The mice were injected with lipopolysaccharide (LPS; intra-peritoneal injection, i.p.) after 24 hr of macrophage reconstitution. Lungs were harvested for evaluation of NLRP3 inflammasome activation and lung inflammation. NLRP3 inflammasome activation (indicated by caspase 1 activation and IL-1β maturation) in the murine lung was assessed by immunoblotting. [file elife-83842-fig6-data1.zip › Figure 6 - source data 1/Figure 6B - Source data for WB labelled.pptx]

## Slide 1
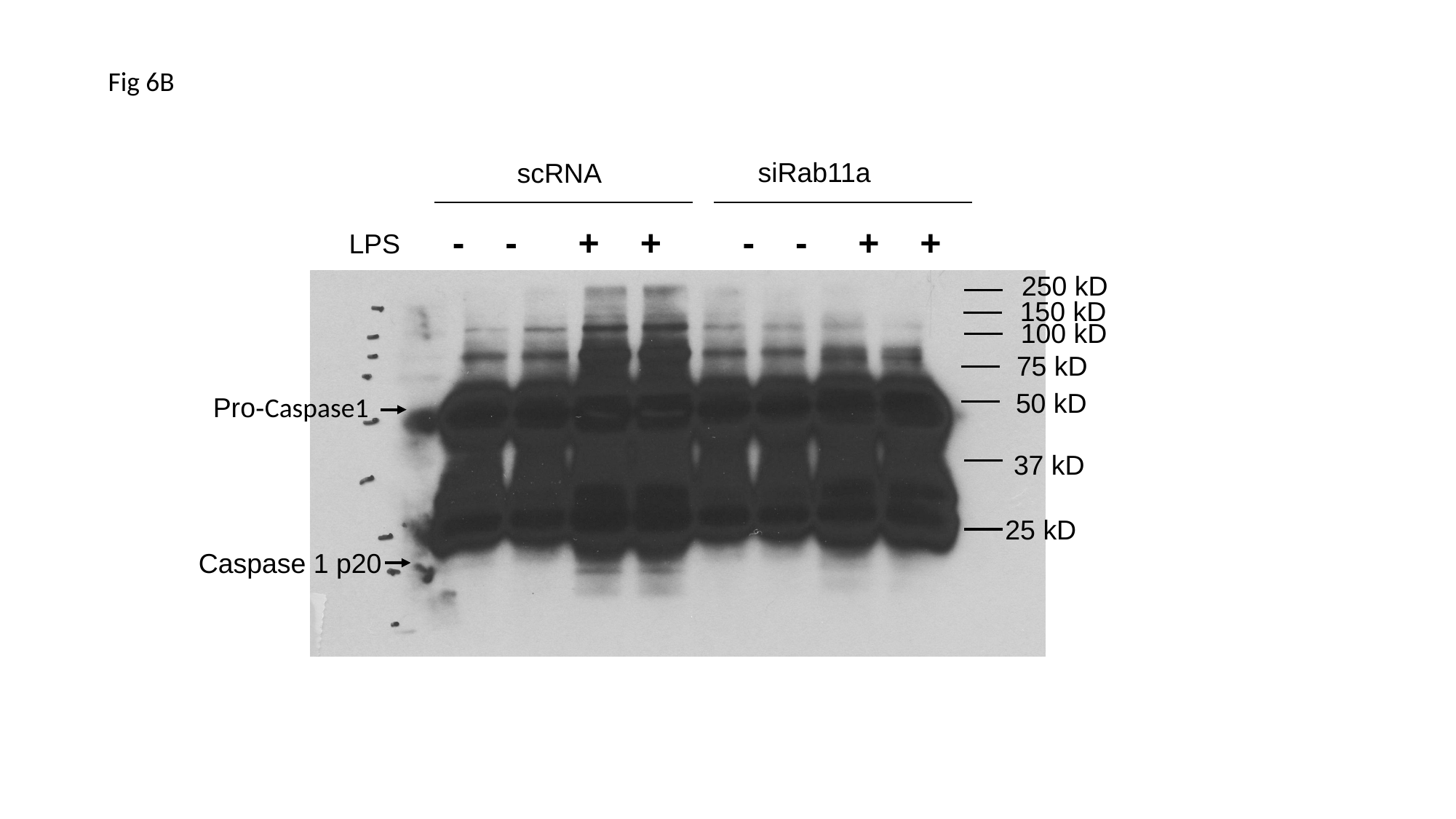

Fig 6B
siRab11a
scRNA
- - + + - - + +
LPS
250 kD
150 kD
100 kD
75 kD
50 kD
Pro-Caspase1
37 kD
25 kD
 Caspase 1 p20

## Slide 2
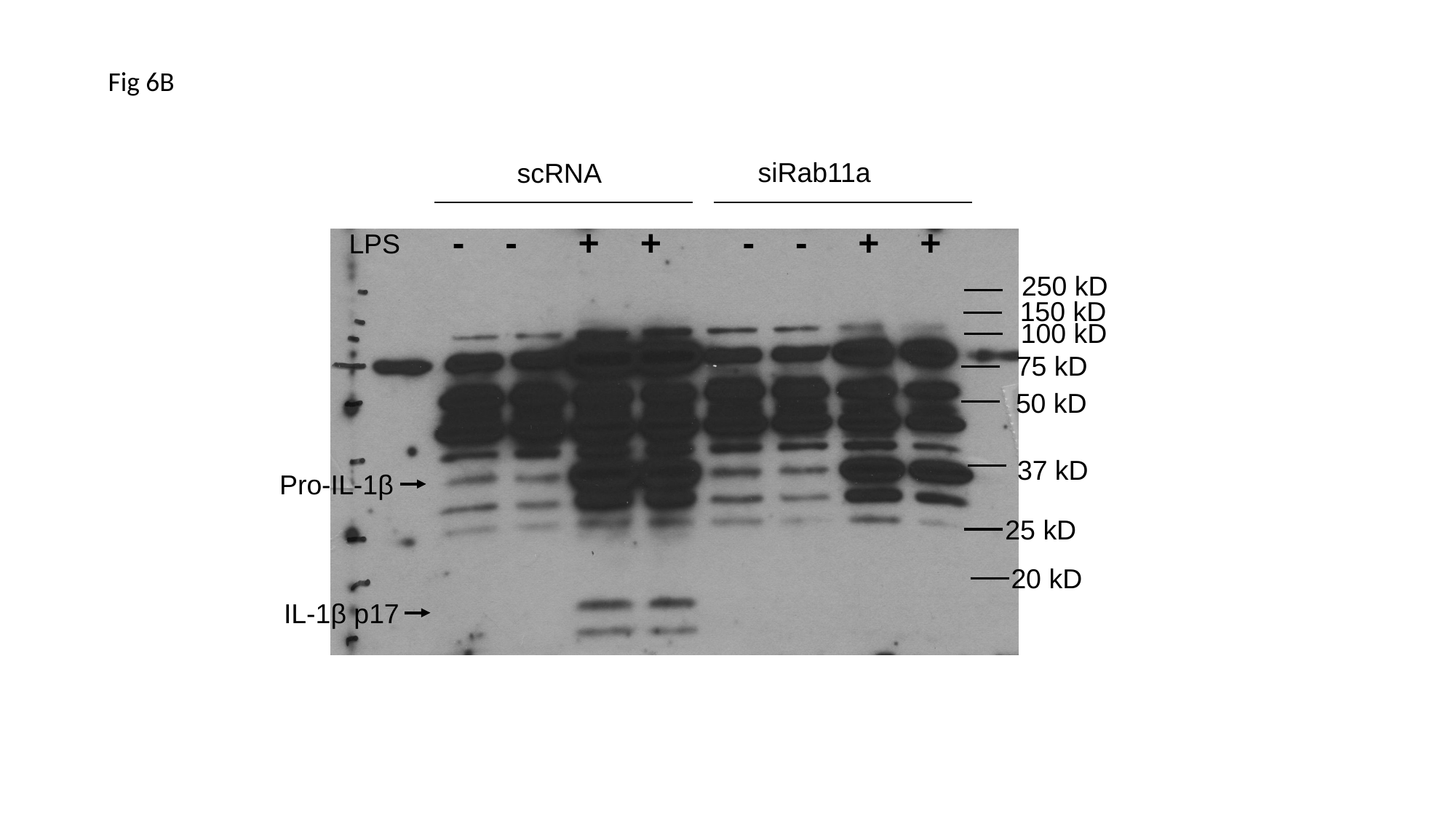

Fig 6B
siRab11a
scRNA
- - + + - - + +
LPS
250 kD
150 kD
100 kD
75 kD
50 kD
37 kD
Pro-IL-1β
25 kD
20 kD
IL-1β p17

## Slide 3
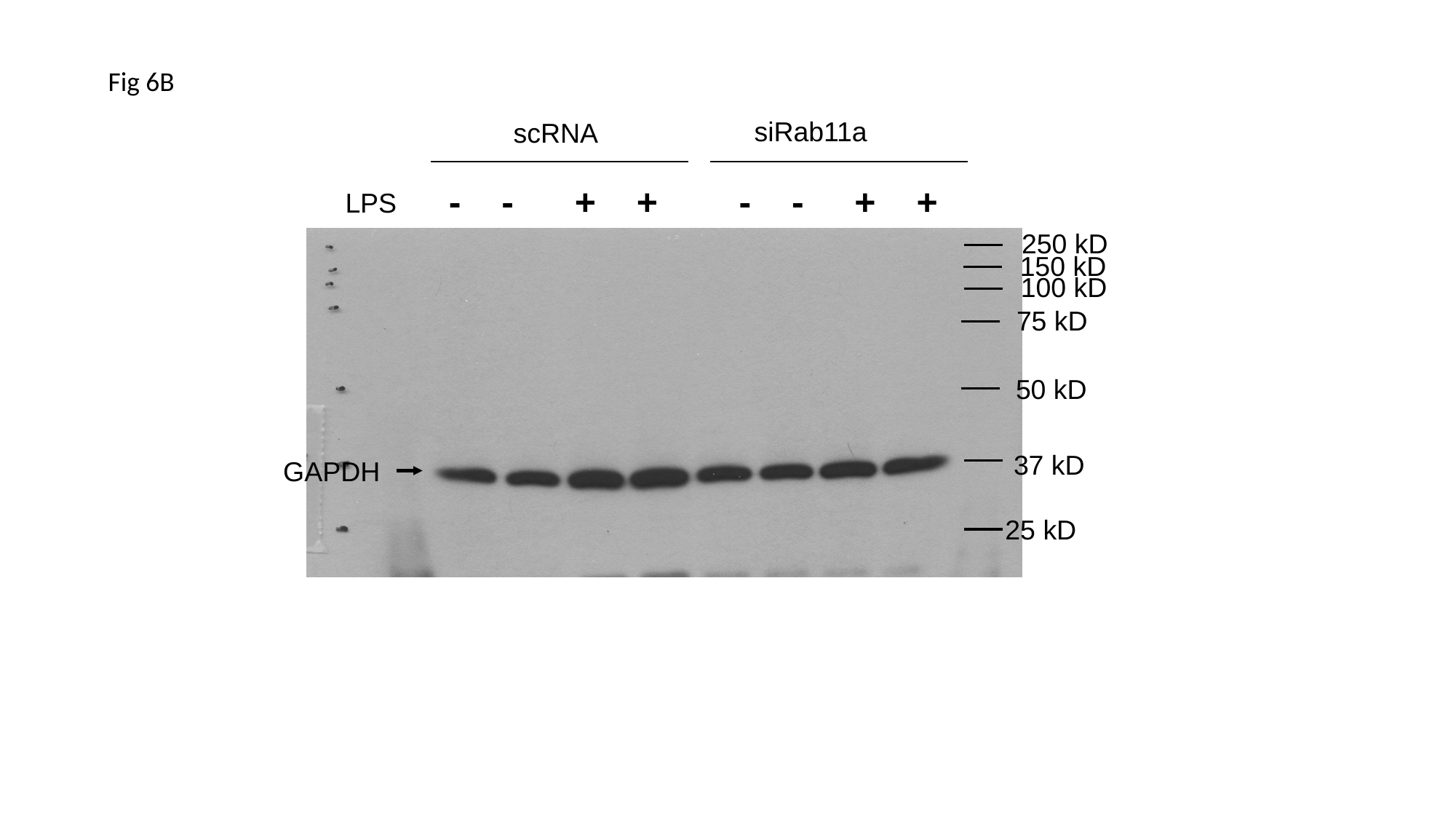

Fig 6B
siRab11a
scRNA
- - + + - - + +
LPS
250 kD
150 kD
100 kD
75 kD
50 kD
37 kD
GAPDH
25 kD
