# Supplementary material for: Endosomal trafficking of two-pore K+ efflux channel TWIK2 to plasmalemma mediates NLRP3 inflammasome activation and inflammatory injury
Source: eLife. 2023 May 9;12:e83842. doi: 10.7554/eLife.83842 (PMC10202452; doi:10.7554/eLife.83842)
Supplement: Figure 6—source data 1. — Related to Figure 6B. Lung macrophages (Mac) were depleted with clodronate liposomes and then reconstituted via intratracheal route with monocyte-derived macrophages (MDMs) treated with either siRNA of Rab11a or siRNA control as illustrated. The mice were injected with lipopolysaccharide (LPS; intra-peritoneal injection, i.p.) after 24 hr of macrophage reconstitution. Lungs were harvested for evaluation of NLRP3 inflammasome activation and lung inflammation. NLRP3 inflammasome activation (indicated by caspase 1 activation and IL-1β maturation) in the murine lung was assessed by immunoblotting. [file elife-83842-fig6-data1.zip › Figure 6 - source data 1/Figure 6B.pptx]

## Slide 1
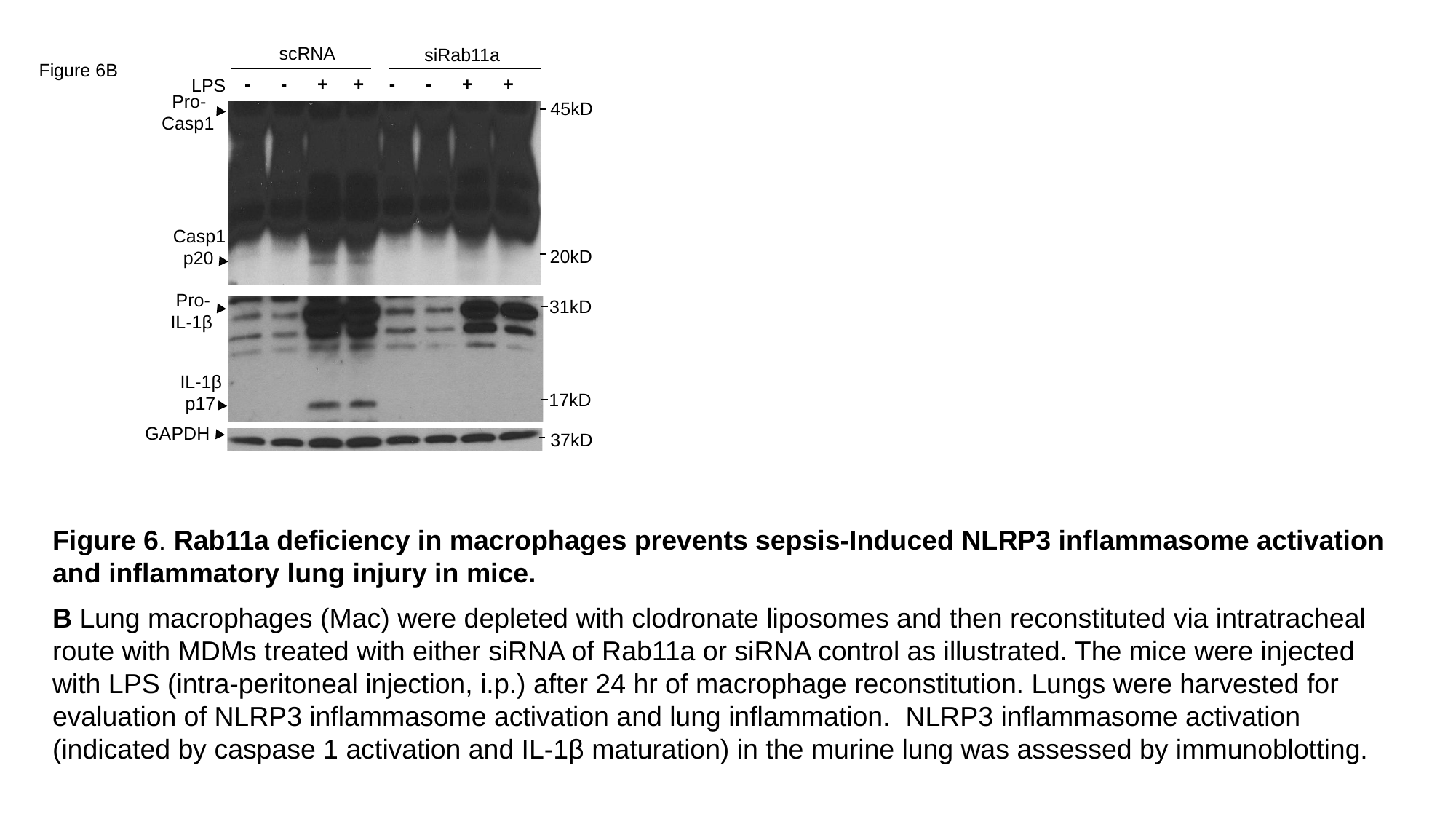

scRNA
siRab11a
- - + + - - + +
LPS
 Figure 6B
 Pro-
 Casp1
45kD
 Casp1
 p20
20kD
 Pro-
 IL-1β
31kD
IL-1β
 p17
17kD
GAPDH
37kD
Figure 6. Rab11a deficiency in macrophages prevents sepsis-Induced NLRP3 inflammasome activation and inflammatory lung injury in mice.
B Lung macrophages (Mac) were depleted with clodronate liposomes and then reconstituted via intratracheal route with MDMs treated with either siRNA of Rab11a or siRNA control as illustrated. The mice were injected with LPS (intra-peritoneal injection, i.p.) after 24 hr of macrophage reconstitution. Lungs were harvested for evaluation of NLRP3 inflammasome activation and lung inflammation. NLRP3 inflammasome activation (indicated by caspase 1 activation and IL-1β maturation) in the murine lung was assessed by immunoblotting.
